# Six-Month Prostate Cancer Empowerment Program (PC-PEP) Improves Urinary Function: A Randomized Trial

**DOI:** 10.3390/cancers16050958

**Published:** 2024-02-27

**Authors:** Tarek Lawen, Gabriela Ilie, Ross Mason, Ricardo Rendon, Jesse Spooner, Emmi Champion, Jessica Davis, Cody MacDonald, Michael J. Kucharczyk, Nikhilesh Patil, David Bowes, Greg Bailly, David Bell, Joseph Lawen, Derek Wilke, George Kephart, Robert David Harold Rutledge

**Affiliations:** 1 Department of Urology, Dalhousie University, Halifax, NS B3H 4R2, Canada; 2 Department of Radiation Oncology, Dalhousie University, Halifax, NS B3H 4R2, Canada; 3 Department of Community Health and Epidemiology, Dalhousie University, Halifax, NS B3H 4R2, Canada; codymacdonald@dal.ca (C.M.);; 4 Department of Oncology, Queens University, Kingston, ON K7L 3N6, Canada; 5 School of Health Administration, Dalhousie University, Halifax, NS B3H 4R2, Canada; 6 School of Nursing, Umeå University, 901 87 Umeå, Sweden

**Keywords:** prostate cancer, pelvic floor muscle training (PFMT), patient education and activation, behavioural intervention, urinary function, incontinence, erectile dysfunction, sexual function, eHealth, cancer survivorship

## Abstract

**Simple Summary:**

In this research, we explore the effectiveness of a unique home-based 6-month comprehensive program designed to empower prostate cancer patients during their treatment. The Prostate Cancer-Patient Empowerment Program (PC-PEP) aims to enhance the quality of life for men undergoing curative treatment for prostate cancer, focusing on improving urologic function. Through a blend of physical activities, dietary education and recommendations, stress management, and social support, PC-PEP offers an innovative approach to patient care. This study rigorously assesses the impact of PC-PEP through a detailed 6-month comparison with standard care, evaluating its potential to significantly improve patient-reported outcomes. Our findings hold the promise of reshaping patient care strategies, presenting a potentially valuable addition to clinical practices for men battling prostate cancer, with the hope of improving not just their physical well-being but also their overall quality of life.

**Abstract:**

**Purpose:** This is a secondary analysis examining a six-month home-based Prostate Cancer-Patient Empowerment Program (PC-PEP) on patient-reported urinary, bowel, sexual, and hormonal function in men with curative prostate cancer (PC) against standard of care. **Methods:** In a crossover clinical trial, 128 men scheduled for PC surgery (n = 62) or radiotherapy with/without hormones (n = 66) were randomized to PC-PEP (n = 66) or waitlist-control and received the standard of care for 6 months, and then PC-PEP to the end of the year. PC-PEP included daily emails with video instructions, aerobic and strength training, dietary guidance, stress management, and social support, with an initial PFMT nurse consultation. Over 6 months, participants in the PC-PEP received optional text alerts (up to three times daily) reminding them to follow the PFMT video program, encompassing relaxation, quick-twitch, and endurance exercises; compliance was assessed weekly. Participants completed baseline, 6, and 12-month International Prostate Symptom Score (IPSS) and Expanded Prostate Cancer Index Composite (EPIC) questionnaires. **Results:** At 6 months, men in the PC-PEP reported improved urinary bother (IPSS, *p* = 0.004), continence (EPIC, *p* < 0.001), and irritation/obstruction function (*p* = 0.008) compared to controls, with sustained urinary continence benefits at 12 months (*p* = 0.002). Surgery patients in the waitlist-control group had 3.5 (95% CI: 1.2, 10, *p* = 0.024) times and 2.3 (95% CI: 0.82, 6.7, *p* = 0.11) times higher odds of moderate to severe urinary problems compared to PC-PEP at 6 and 12 months, respectively. **Conclusions:** PC-PEP significantly improves lower urinary tract symptoms, affirming its suitability for clinical integration alongside established mental health benefits in men with curative prostate cancer.

## 1. Introduction

Prostate cancer is among the most common cancers affecting men worldwide, with approximately 1.4 million cases and over 375,000 deaths annually [1]. The mainstay radical treatments for localized prostate cancer include radical prostatectomy and external beam radiotherapy. Both treatment modalities confer similar oncologic outcomes, providing patients with durable survivorships [2]. Due to the high cure rates for prostate cancer, patients may experience long-term complications related to the treatment [3,4]. 

Urinary incontinence (UI) affects 69–98% of men undergoing radical prostatectomy [5] and 1–10% undergoing radiation therapy [6]. UI can be emotionally distressing and debilitating, with some men rating it as a more bothersome outcome than erectile dysfunction [7,8]. Men may socially withdraw, ultimately leading to anxiety, depression, and marital issues [9]. As such, interventions aimed at reducing the risk of and accelerating the recovery from this feared complication are integral in the care of our prostate cancer patients. 

Pelvic floor muscle training (PFMT) is an effective, non-invasive, and safe treatment for UI [10]. PFMT, through targeted repetitive contractions, primarily strengthens the striated urethral sphincter, bulbocavernosus, and puborectalis—a component of the levator ani. This specific focus enhances urinary continence mechanisms in men, as extensively detailed in the body of work by Stafford and Hodges [11,12,13]. Consequently, PFMT increases the endurance and strength of the pelvic floor muscles, aiming to improve urinary, bowel, and sexual symptoms [11,12,13,14].

Randomized trials have examined PFMT’s effectiveness in managing post-radical prostate cancer treatment UI, yielding conflicting results [15]. Some studies support PFMT in reducing UI, while others suggest that UI may improve over time regardless of intervention [16,17]. These disparities may arise from variations in incontinence definitions, assessment tools, PFMT regimens, and compliance with the protocols [17]. PFMT programs that commence pre-operatively continue for several months post-operatively and target both fast- and slow-twitch muscle groups with progressive difficulty have not been adequately studied. 

The 6-month Prostate Cancer Patient Empowerment Program (PC-PEP) is a home-based intervention aimed at addressing the biopsychosocial needs of prostate cancer patients prior to, during, and after treatment [4]. The PC-PEP program utilizes a multi-faceted online and live, interactive approach to patient empowerment that includes aerobic and strength exercise, PFMT, meditation with a biofeedback diet, and social support to improve overall health and treatment-related outcomes in prostate cancer patients. The outcomes of the PC-PEP trial, which involved the comparison of mental health outcomes in 128 men scheduled for curative treatment of prostate cancer, revealed that individuals who underwent the PC-PEP had lower rates of psychological distress necessitating clinical intervention compared to men who received the standard of care [4]. Here, we conduct secondary outcomes analyses to evaluate the program’s impact on patient-reported urinary, bowel, and sexual function symptoms.

## 2. Materials and Methods

In this single-center crossover randomized clinical trial, 171 men residing in Halifax, Nova Scotia, were assessed for eligibility, referred by urologists, radiation oncologists, or through self-referral between December 2019 and January 2021. The study protocol has been previously published [4]. Inclusion criteria required participants to be older than 18 years of age, diagnosed with biopsy-confirmed prostate adenocarcinoma, scheduled for curative prostate cancer treatment (including radical prostatectomy or salvage radiotherapy with or without hormone therapy) within 6 months of trial randomization, capable of participating in low to moderate exercise regimens, proficient in English, willing to travel to Halifax, Nova Scotia for in-person physical assessments at baseline, 6, and 12 months, and able to access their email daily. Interested individuals provided informed consent following institutional Nova Scotia Health Authority approval (ClinicalTrials.gov NCT03660085). The study adhered to the CONSORT reporting guideline [4]. 

**Figure 1:**
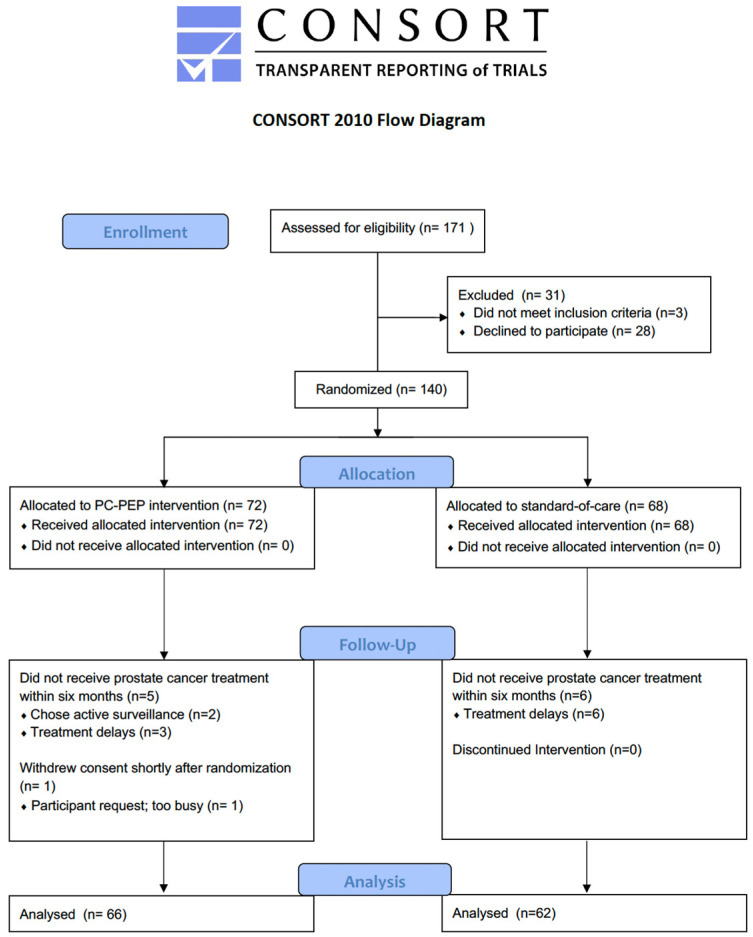
CONSORT 2010 flow diagram. CONSORT = Consolidated Standards of Reporting Trials; PC-PEP = Prostate Cancer Patient Empowerment Program.

Out of the initial 171 eligible men, 140 were randomized. Twelve patients were subsequently excluded, including one who withdrew consent and eleven who did not receive curative treatment within 6 months. Of the 128 patients who participated in the trial, 62 patients were scheduled for radical prostatectomy (58 robot-assisted) and 66 for radiotherapy (Figure 1). 

Radical prostatectomy patients underwent the procedure at a median of 61 days post-randomization, while radiotherapy patients commenced at a median of 73 days post-randomization, with interquartile ranges of 33–99 and 29–101 days, respectively. The randomization allocation table was securely stored in an Excel file protected by a password, with access restricted solely to the principal investigator (PI), who was not part of the consent or assessment procedures. The randomization process remained concealed from patients, clinicians, and research staff. Once the patient had completed all preliminary assessments, the PI used the allocation sequence from the table to assign them to either the intervention or control group. Patients were randomized (1:1) to either the PC-PEP intervention or the control wait-list (standard of care) for the first 6 months post-treatment. 

Patients were assigned to the intervention or control group based on a computer-generated fixed block randomization allocation scheme, aiming to balance baseline covariates. Consistent with the prevailing standard of care within Nova Scotia, all patients undergoing radical prostatectomy were referred to the Urology Clinic’s dedicated pelvic floor nurse specialist for a customized assessment and pelvic floor musculature training regimen, initiated 3–4 weeks after the surgical intervention by the urologist. This protocol mirrors the outpatient care paradigm adopted regionally, underscoring the critical role of pelvic floor physiotherapy in the postoperative recovery trajectory of prostatectomy patients. In contrast, individuals receiving radiotherapy were not accorded such referrals, aligning with the differentiated post-treatment support framework predicated on the modality of the primary treatment administered. A total of 66 patients were randomized to PC-PEP and 62 to the wait-list control group. At 6 months, a crossover occurred, with the wait-list control group receiving the PC-PEP intervention while the intervention group retained access to PC-PEP materials and live online monthly video conferences until the trial’s end (12 months post-randomization).

Patients completed the International Prostate Symptom Score (IPSS) and Expanded Prostate Cancer Index Composite (EPIC) questionnaires to evaluate urinary, bowel, and sexual function, as well as other prognostic covariates, at baseline, 6, and 12 months [4]. Biometric data were measured in person, while medical charts were reviewed at the trial’s conclusion.

### 2.1. Exposure

The PC-PEP intervention is outlined in the study protocol, available elsewhere. 4 Patients received daily emails during the six-month intervention, featuring a 3–5-minute video by co-authors GI and RDHR. These videos provided education, motivation, and prescribed physical, mental, and social activities. Patients were encouraged to exercise daily, perform PFMT three times daily, and engage in daily relaxation using a biofeedback device (HeartMath^®^, Boulder Creek, CA, USA). The program was customized to each patient’s fitness level and included guidance on diet, sleep hygiene, vitamin D intake, intimacy, sexuality, erectile dysfunction, and communication techniques. Men had the option to call two other participants weekly and join monthly Zoom conferences led by co-authors GI and RDHR.

The PFMT in PC-PEP began with a 20–30-minute initial in-person training session by a urology pelvic floor nurse, followed by a 20-minute educational video by nurse PFMT specialists. Every Sunday, participants received a weekly 7–9-minute video outlining the PFMT routine to be done three times daily. On 16 of the 26 Sundays, a 5-minute video explained the week’s routine and encouraged proper technique. Daily emails included the PFMT video link and practice reminders, with optional text reminders, with the link, at 9 am, 2 pm, and 7 pm.

Each PFMT video, delivered by co-author RR, followed a sequence based on expert advice from pelvic floor physiotherapists [18,19,20]. The instructional regimen comprised the following components: (a) a two-minute segment dedicated to a mindfulness-based relaxation technique, focusing on fostering abdominal breathing and the relaxation of the pelvic floor; (b) execution of ten ‘Long Holds’, involving contractions sustained for ten seconds followed by a ten-second relaxation period; (c) performance of ten ‘Quick Flicks’, featuring swift contractions and relaxations at a frequency of 90 contractions per minute; (d) sequence of 10 ‘Long Holds’; (e) a repetition of ten ‘Quick Flicks’; (f) implementation of the ‘Blow before you go’ technique, carried out twice. This technique entailed holding the breath at the end of an exhalation, contracting and holding the pelvic floor muscles, engaging in a high-risk activity such as squatting, and then returning to a resting state. The Supplementary Materials include details regarding each week’s instructional video.

### 2.2. Primary Outcomes 

Lower urinary tract symptoms were assessed using the well-validated International Prostate Symptom Score (I-PSS), a commonly employed clinical tool [21]. The questionnaire covered symptoms such as incomplete bladder emptying, urination frequency, intermittent urination, urgency, weak urinary stream, straining during urination, and nocturia. Responses were graded from 0 (not at all) to 5 (almost always), with higher scores indicating more severe symptoms. Both continuous and binary variables were assessed (coded 0 for mild urinary symptoms/sum IPSS scores between 0 to 7 and 1 for moderate to severe urinary symptoms/sum scores between 8 and 35). 

The Expanded Prostate Cancer Index Composite (EPIC) was used to assess Urinary, Bowel, Hormonal, and Sexual Function [22,23]. There were four items for the ‘urinary incontinence’ and ‘urinary irritative/obstructive’ domains, five items for the ‘hormonal’ domain, and six items each for the ‘bowel’ and ‘sexual’ domains. The scores range from 0–100, with higher scores indicating better function. 

### 2.3. Pelvic Floor Compliance

Weekly adherence to the pelvic floor exercise regimens in the PC-PEP program was monitored using patient-reported compliance surveys. These surveys recorded the frequency of completing the prescribed PFMT activity (3 times a day, 8.77 min per session) and the total daily PFMT duration (in minutes) reported by participants for that week.

### 2.4. Prognostic Covariates

Prognostic covariates included patient age (years) [24], Charlson Comorbidity Index [25,26], days between randomization and treatment start [27], and treatment modality (surgery ‘1’, primary radiotherapy/salvage radiotherapy ‘2’) and were determined a priori [28,29].

The sample size for the PC-PEP RCT was determined based on the primary outcome of the trial, which focused on assessing psychological distress and the need for clinical treatment, as previously detailed in another publication [4]. Due to the predefined nature of the trial’s primary outcome, no separate sample analysis for the current outcome was conducted.

### 2.5. Statistical Analysis

Median or count comparisons between the two study arms on demographics, cancer-related, and health-related characteristics were used to assess baseline differences between the two groups computed using Mann–Whitney’s U test and Fisher’s exact test, respectively. Two-level linear modeling (for group and treatment type stratified analyses) was used to assess the fixed effects of the group (PC-PEP vs. control) over time (baseline, 6-month) on I-PSS and EPIC continuous scores with prognostic covariates controlled, incorporating restricted maximum likelihood (REML) estimation [28,29]. Cross-tabulation analyses were conducted to examine the relationship between the binary I-PSS variable by group. Logistic regression was used to analyze the 6-month binary I-PSS by group assignment, prognostic covariates, and baseline I-PSS scores. Pelvic floor weekly compliance over time (26 weeks) for the early versus late/waitlist-control intervention groups was assessed using generalized linear mixed modeling (GLMM) using GENLINMIXED procedure in SPSS with a random intercept for subject and random slope for time and assessed the time × group (early vs. late/waitlist-control intervention) interaction with both time and group added to the model as fixed factors incorporating REML. The distribution of the PFMF compliance outcomes was set to binomial with a LOGIT link. Analyses were set at *p* < 0.05 (2-sided). Analyses were conducted using IBM SPSS (Armonk, NY, USA) statistical software version 27.0 [30]. 

## 3. Results

No adverse events or missing data occurred during the trial. Demographic and patient characteristics were similar between the PC-PEP and control arms pre-intervention (Table 1). 

Among patients, 78% chose daily text alerts (85% in the early group and 71% in the late/waitlist-control group) as reminders for pelvic floor exercises. Out of the 62 RP patients referred for a post-operative appointment with a PFMT-qualified nurse as part of standard care at 3–4 weeks post-surgery, only 32% (5 in PC-PEP and 15 in waitlist-control) attended. 

**Figure 2:**
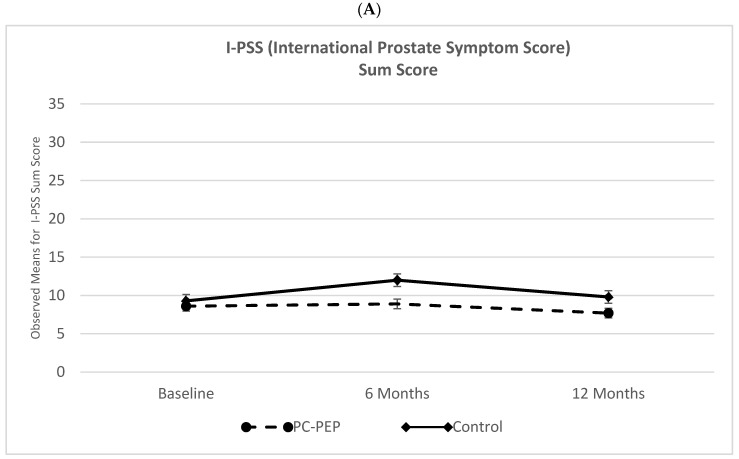

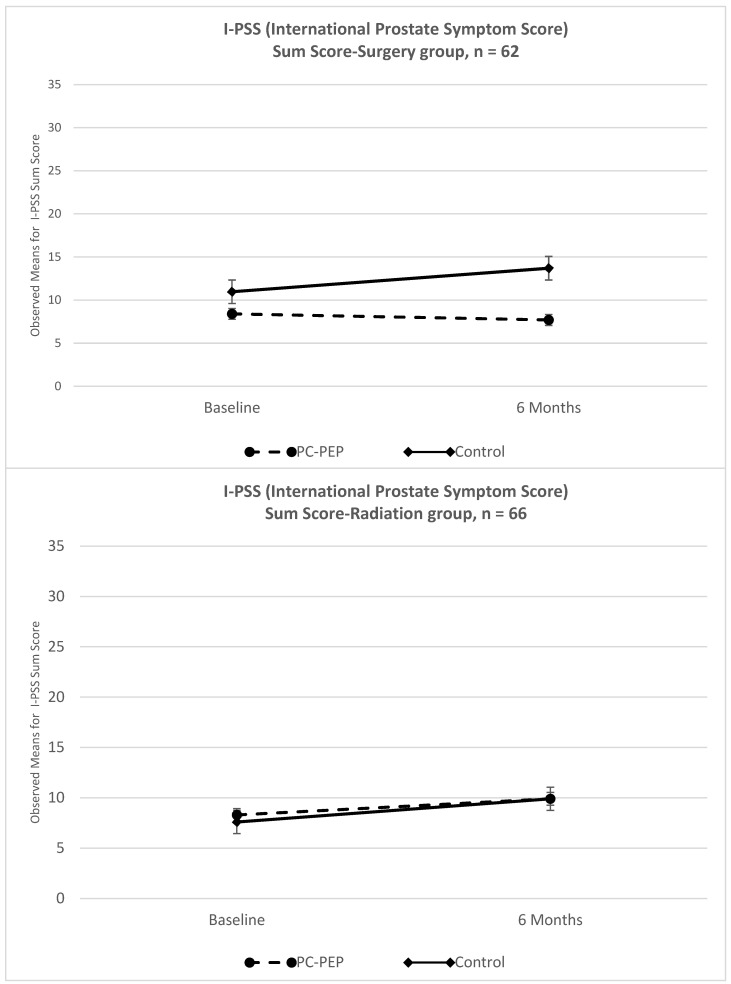

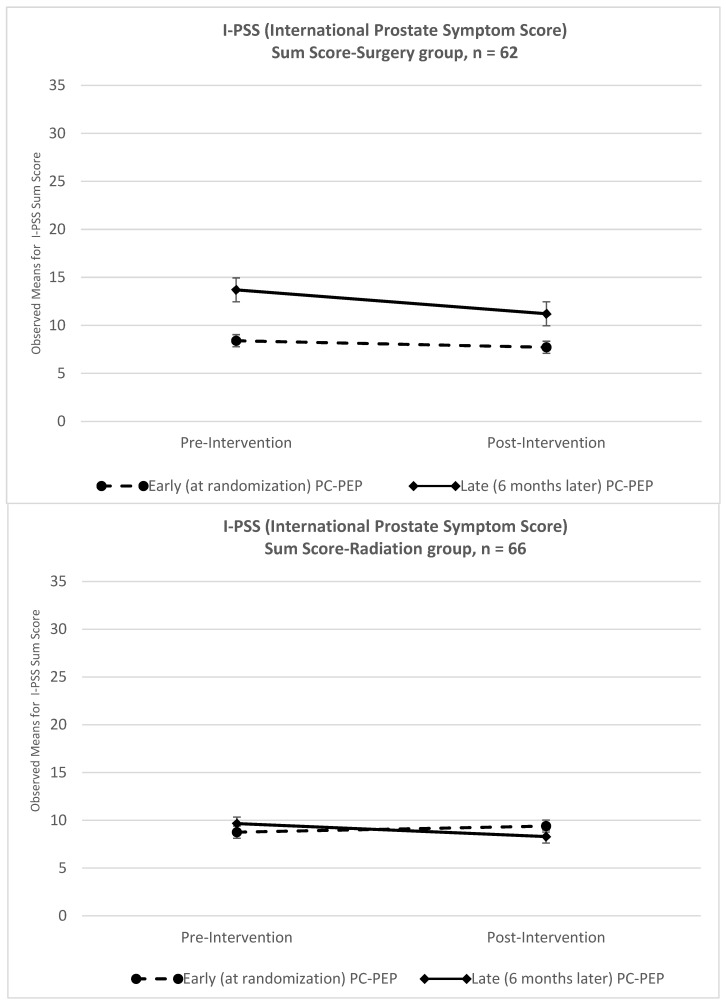

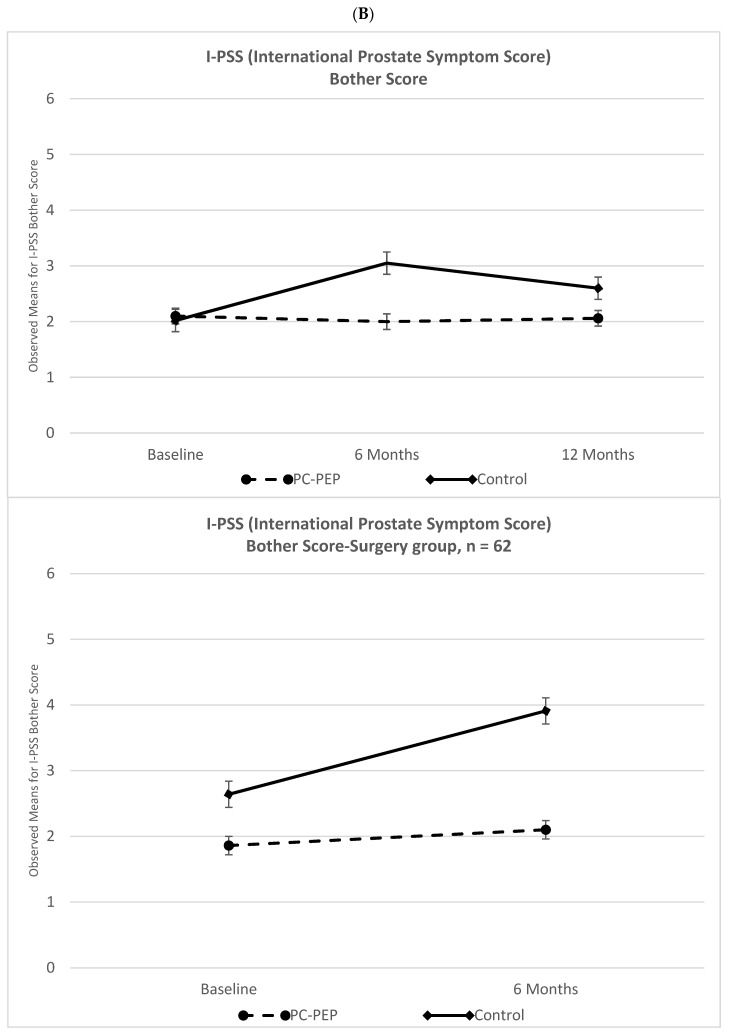

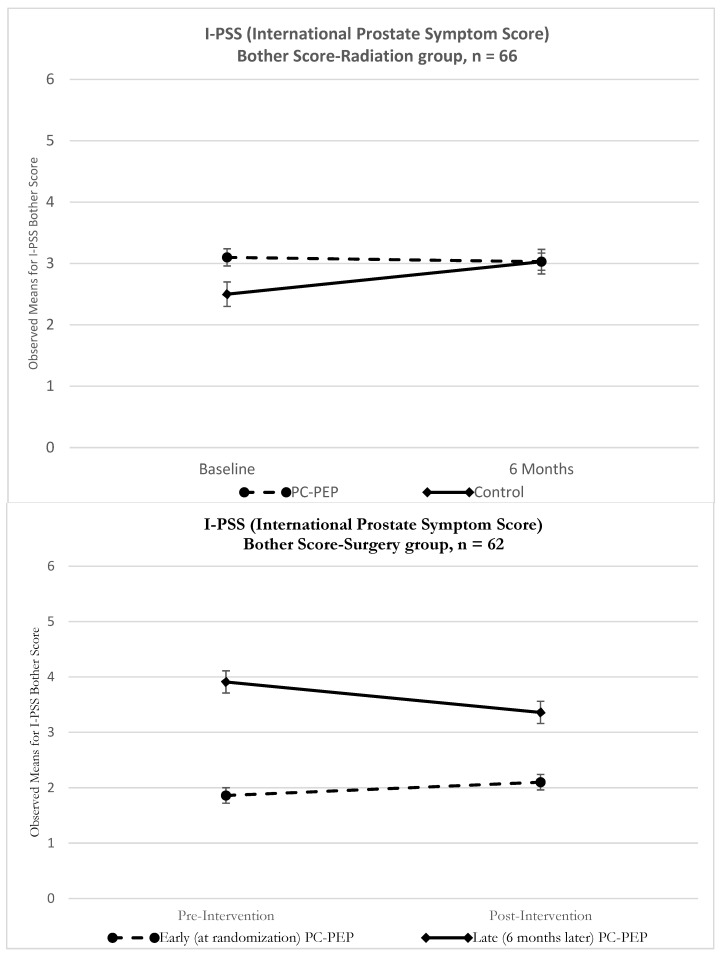

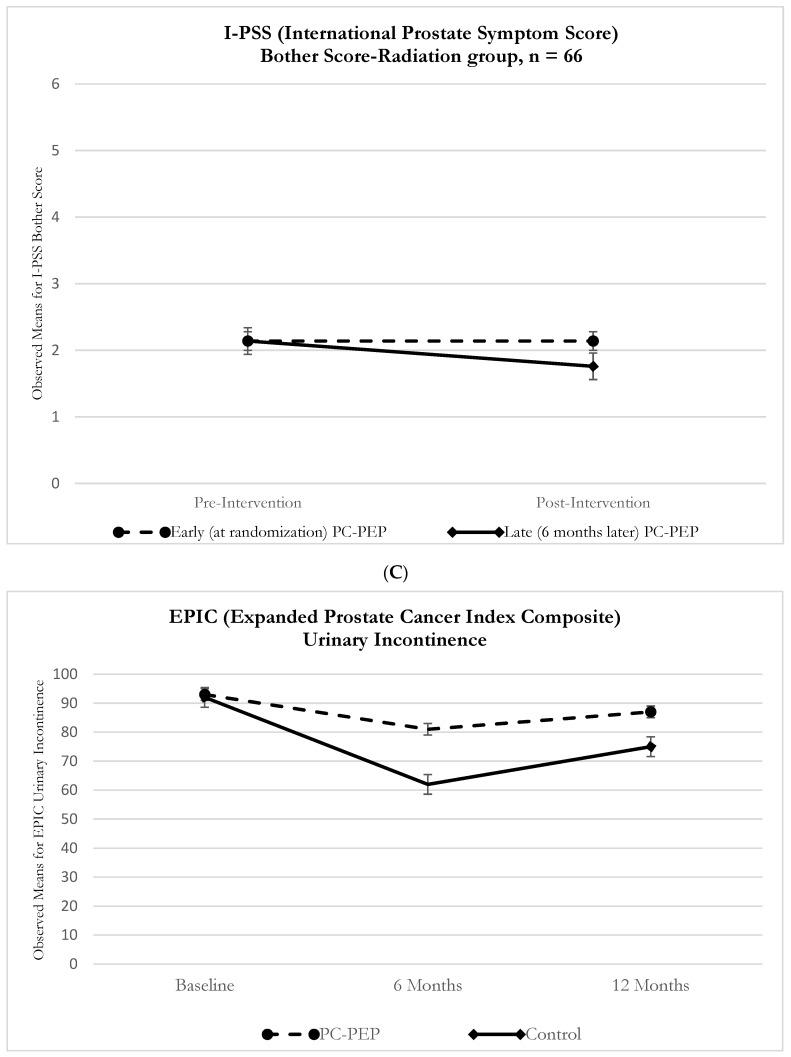

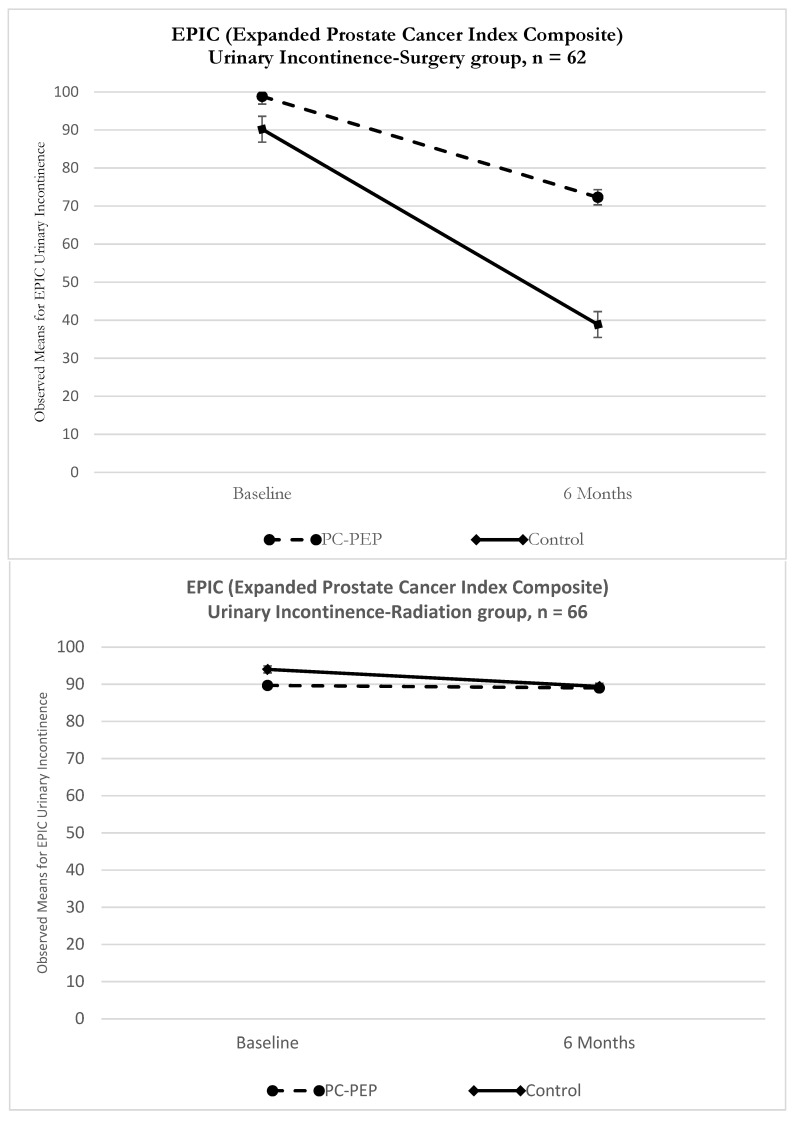

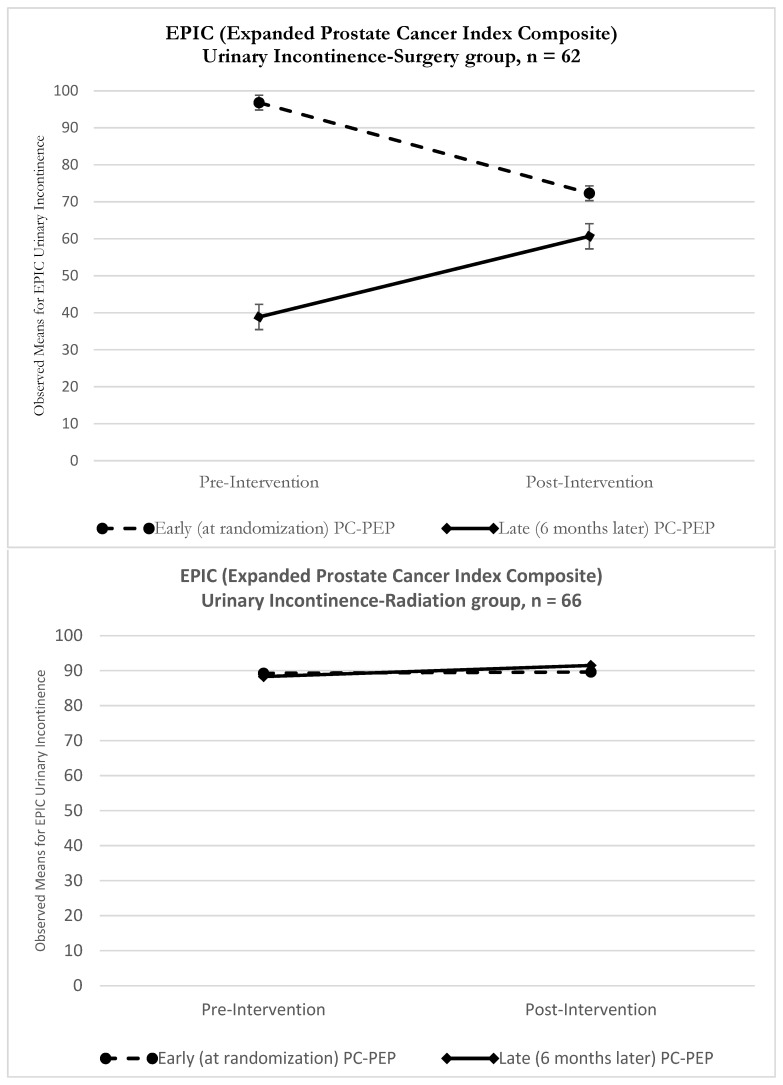

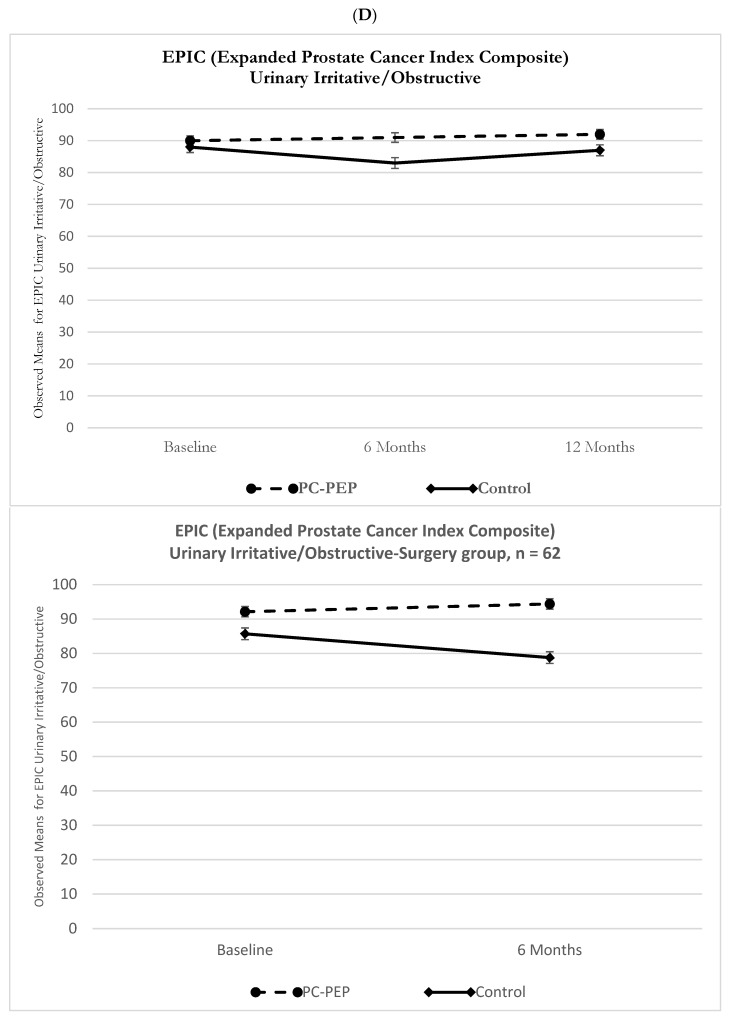

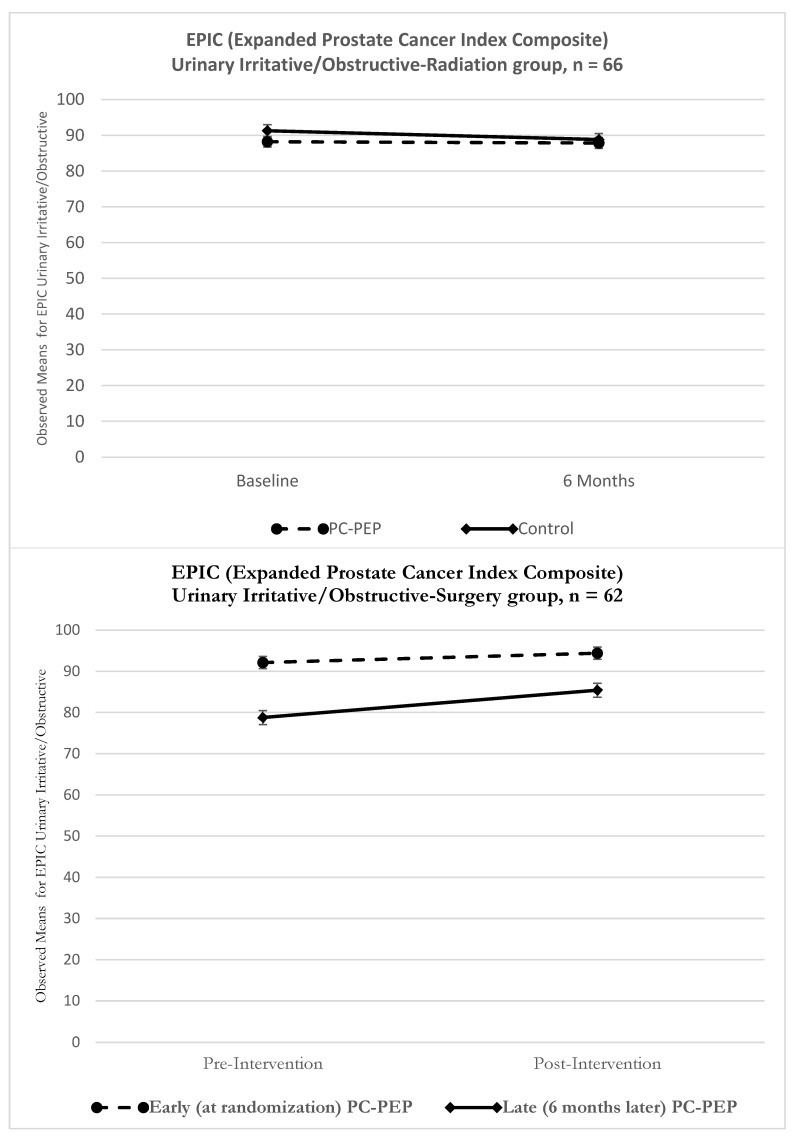

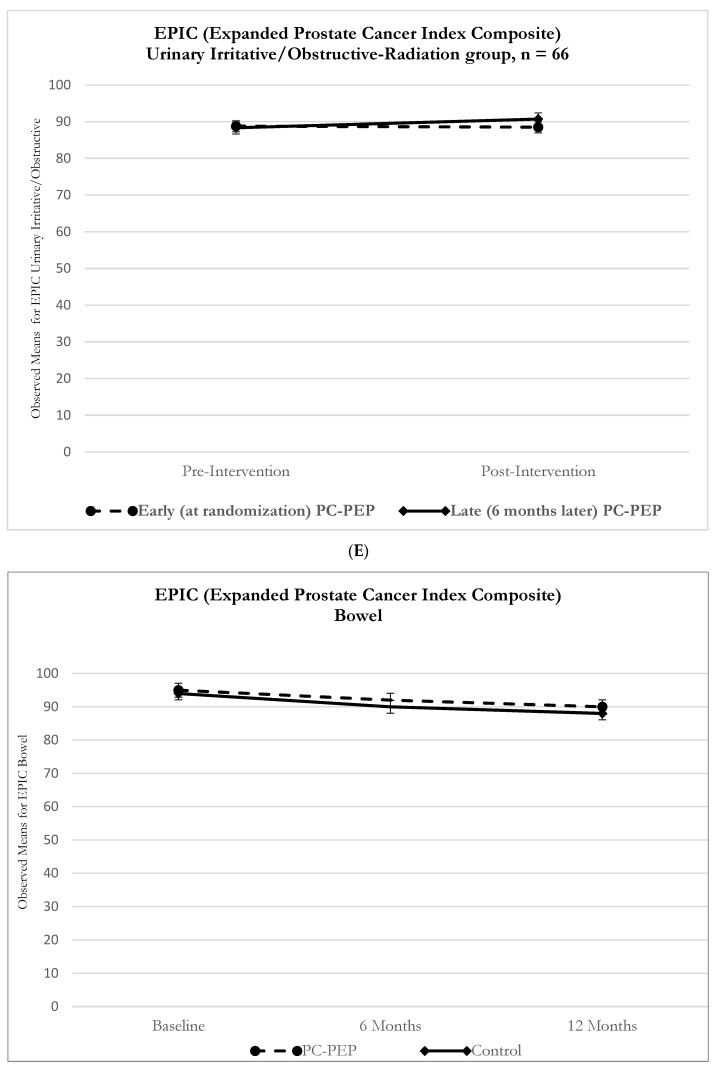

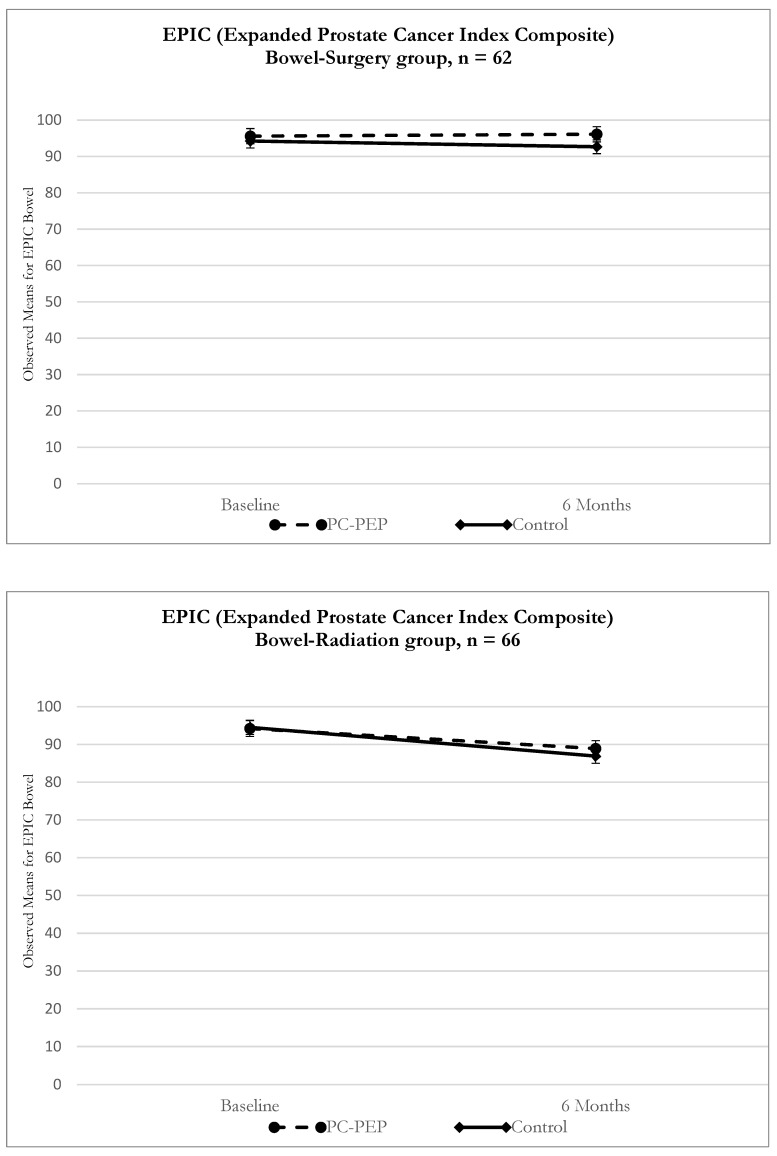

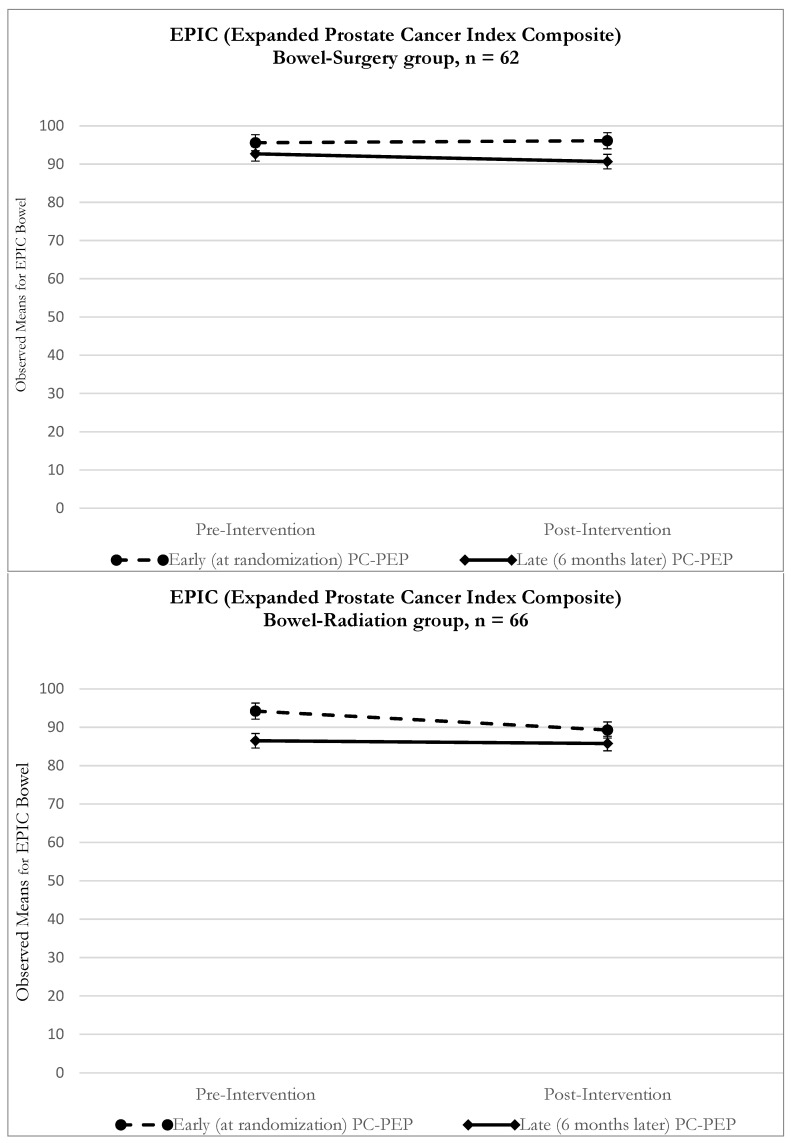

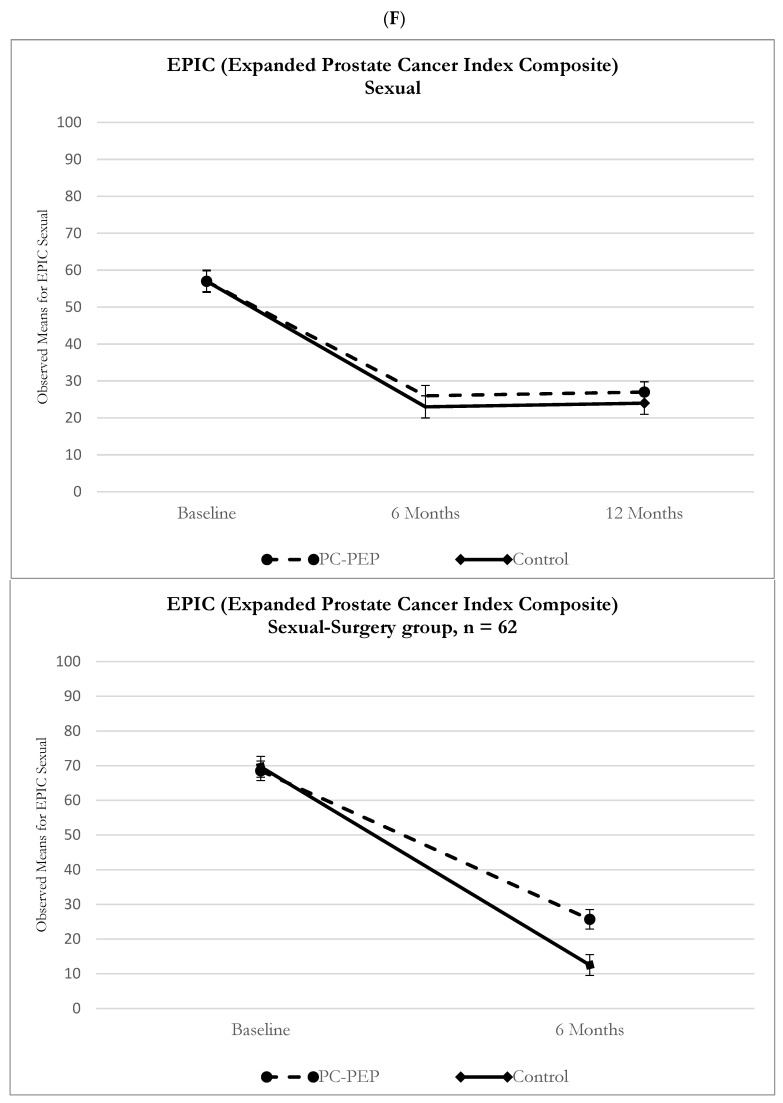

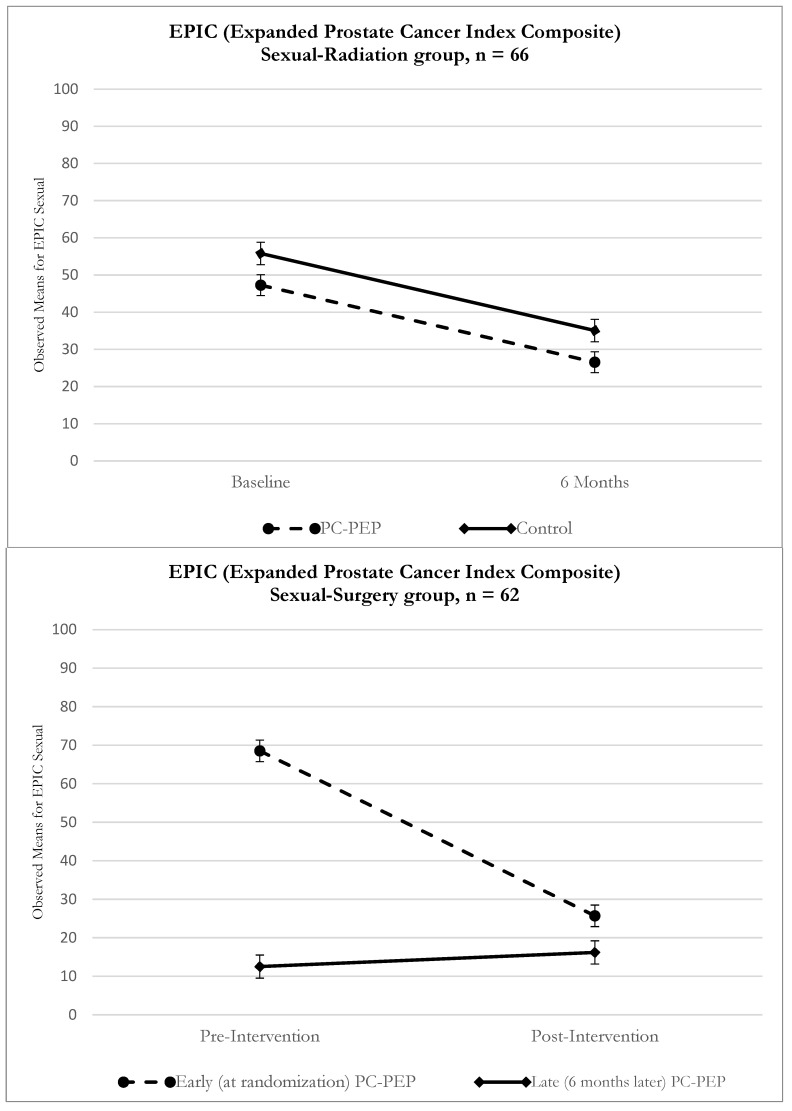

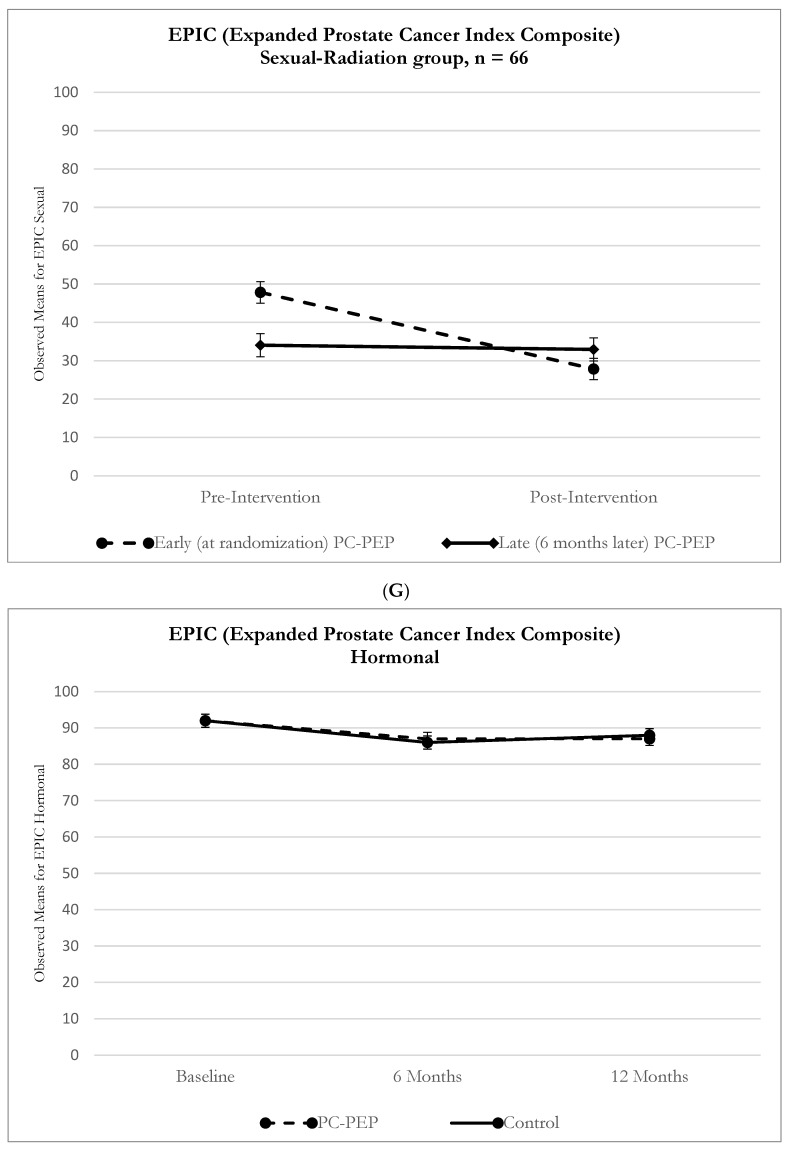

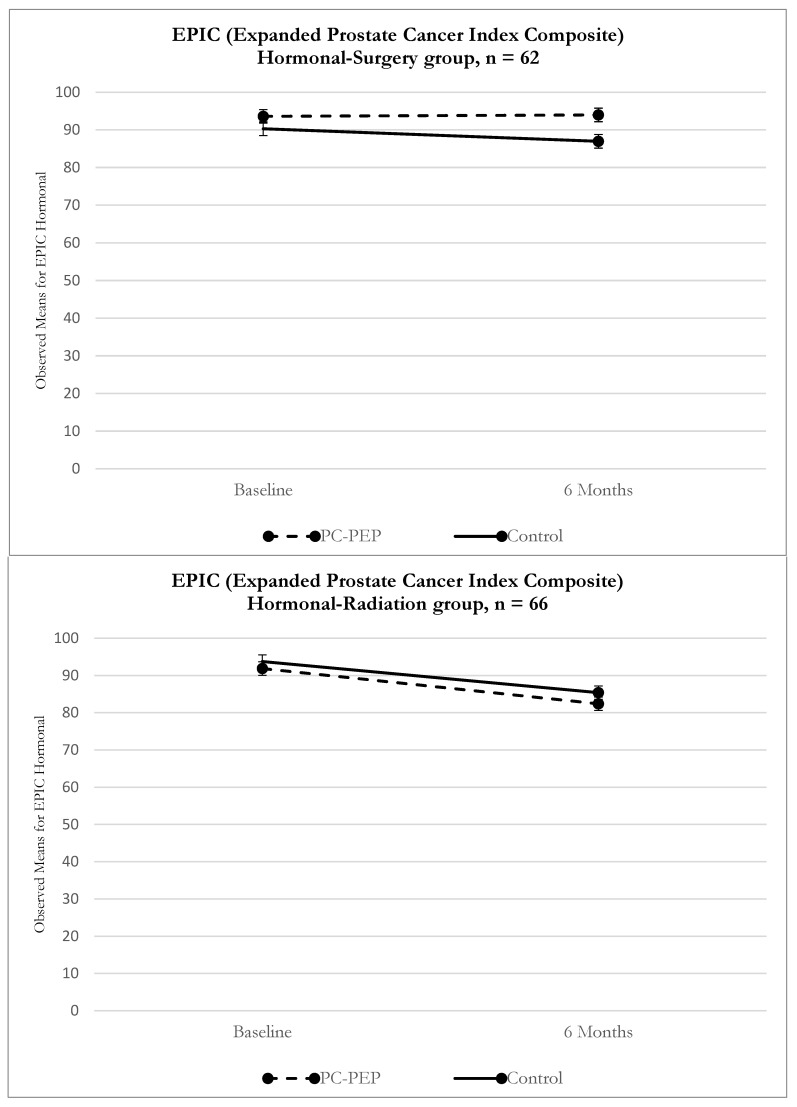

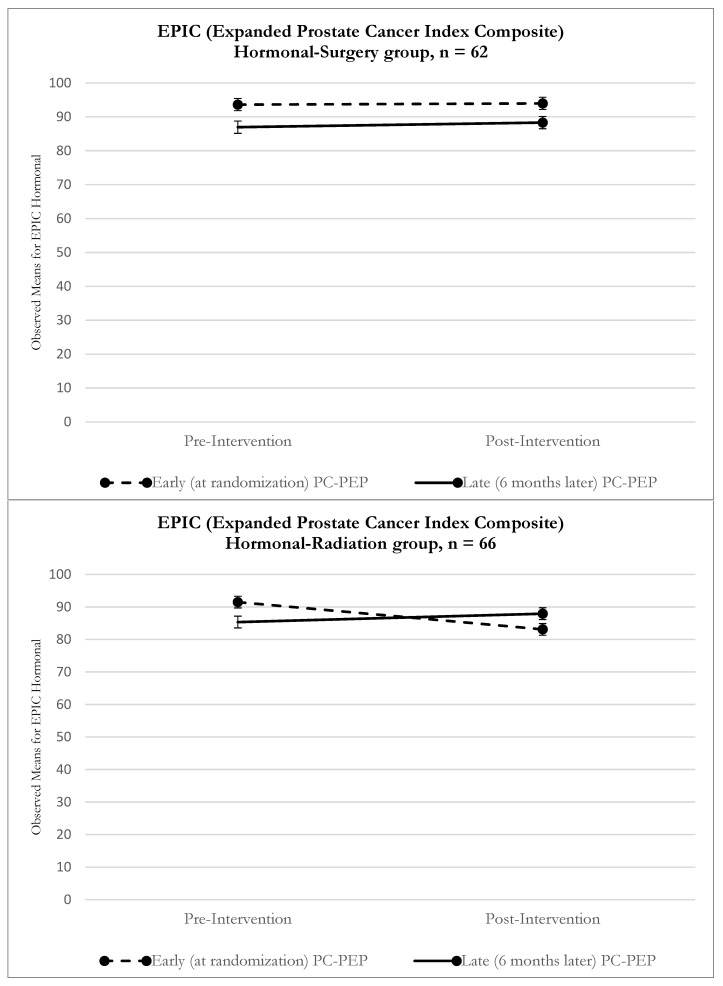
Comparative Analysis of Outcome Measures in Prostate Cancer Patients. This Figure presents a detailed comparison of outcome measures between the control group and participants in the Prostate Cancer Patient Empowerment Program (PC-PEP) across three time points: baseline, 6 months, and 12 months. The data encompass observations from 128 patients who received curative treatment for prostate cancer in Nova Scotia, Canada. Specific measures include (**A**) I-PSS Sum Scores: Average scores on the International Prostate Symptom Score (IPSS), reflecting overall prostate-related symptoms. (**B**) I-PSS Bother Scores: Average scores detailing the level of inconvenience or distress caused by prostate symptoms. (**C**) EPIC Urinary Incontinence Scores: Scores from the Expanded Prostate Cancer Index Composite (EPIC) questionnaire assessing the impact of treatment on urinary incontinence. (**D**) EPIC Urinary Irritative/Obstructive Scores: EPIC scores evaluating symptoms of urinary irritation or obstruction. (**E**) EPIC Bowel Scores: EPIC assessments of bowel function and related quality of life issues. (**F**) EPIC Sexual Function Scores: Scores reflecting the impact of treatment on sexual health and functioning. (**G**) Hormonal Scores: Measures evaluating the effects of treatment on hormonal health and related symptoms. Subsequent figures provide a more granular view, comparing outcomes from baseline to 6 months and analyzing differences between early intervention and late/waitlist-control within the PC-PEP group, stratified by type of treatment received. I-PSS = International Prostate Symptom Score; EPIC = Expanded Prostate Cancer Index Composite; PC-PEP = Prostate Cancer Patient Empowerment Program.

In this study, patients were randomized in a 1:1 ratio to either participate in the Prostate Cancer-Patient Empowerment Program (PC-PEP) intervention or to be placed on a control wait-list, receiving standard of care for the first six months post-operation. At the six-month milestone, a crossover occurred in the design. Patients in the wait-list control group commenced their participation in the PC-PEP intervention, while those initially in the intervention group continued to have access to PC-PEP materials. Additionally, the initial intervention group participated in live online monthly video conferences until the end of the trial period, which was 12 months post-randomization. Figure 2 displays the observed mean values and standard errors of secondary outcomes at baseline, 6, and 12 months for both the early PC-PEP and waitlist-control (late/waitlist-control PC-PEP) patient groups across the trial.

This Figure includes treatment subgroup comparisons between the waitlist control and PC-PEP groups from 0 to 6 months, as well as pre- vs. post-treatment comparisons for both early and late (waitlist-control who received the intervention at 6-months post-trial start) PC-PEP groups (only for illustrative purposes) (the Supplementary Materials provide pre- to post-intervention comparisons for both the early and late/waitlist-control PC-PEP groups. It is essential to approach these results with caution due to inherent differences between the groups. Specifically, the late/waitlist-control intervention group initiated PFMT after completing their treatment, while the early intervention group started PFMT prior to and during their treatment. These differing timelines introduce systematic error and pose challenges for direct comparisons. Moreover, it is important to acknowledge that variations in adherence, engagement, and response to PFMT may have occurred between the two groups, stemming from disparities in treatment initiation. These factors contribute to the complexity of drawing definitive comparisons between the groups).

Two-level linear modeling, controlling for prognostic covariates, showed significant differences between the PC-PEP and waitlist-control groups from baseline to 6 months. Significant improvements were observed in the PC-PEP group for IPSS bother (*p* = 0.004), EPIC urinary incontinence (*p* < 0.001), and EPIC urinary irritative/obstructive (*p* = 0.008) scores (Table 2). However, no significant differences were observed for IPSS urinary symptoms (*p* = 0.059), EPIC bowel (*p* = 0.3), sexual (*p* = 0.4), or hormonal function scores (*p* = 0.6). Supplementary Materials present unadjusted analyses. 

Logistic regression analysis was employed to evaluate the occurrence of moderate to severe urinary problems among patients in the Prostate Cancer-Patient Empowerment Program (PC-PEP) intervention compared to those receiving standard care, with an emphasis on identifying clinically significant differences. This analysis factored in baseline scores and prognostic covariates to ensure a comprehensive evaluation. At the 6-month mark, 55% of patients in the PC-PEP group and 68% of patients receiving standard care reported moderate to severe urinary problems, a notable increase from baseline rates of 46% and 32%, respectively. The odds ratio (OR) for this comparison was 1.7 (95% Confidence Interval [CI]: 0.79, 3.5, *p* = 0.18). A more detailed subgroup analysis based on treatment type revealed differing outcomes. Among surgery patients, 41% in the PC-PEP group and 73% receiving standard care reported moderate to severe urinary problems at 6 months. This is compared to baseline rates of 52% and 67%, respectively, with an OR of 3.5 (95% CI: 1.2, 10, *p* = 0.024). Conversely, in the radiation therapy group, 65% of PC-PEP patients and 62% receiving standard care reported such problems at 6 months, compared to 46% and 45% at baseline, with an OR of 0.63 (95% CI: 0.21, 1.9, *p* = 0.4).

By the 12-month evaluation, 46% of patients in the PC-PEP group and 53% in the standard care group reported moderate to severe urinary problems, which was a return to baseline levels of 46% and 32%, respectively (OR = 1.4, 95% CI: 0.69, 2.9, *p* = 0.34). A 12-month subgroup analysis showed that among surgery patients, 41% in the PC-PEP group and 64% in the standard care group reported moderate to severe urinary problems, a change from baseline rates of 52% and 67%, respectively (OR = 2.3, 95% CI: 0.82, 6.7, *p* = 0.11). In the radiation therapy group, 49% of PC-PEP patients and 45% receiving standard care reported such problems at 12 months, compared to baseline rates of 46% and 45%, with an OR of 0.82 (95% CI: 0.29, 2.3, *p* = 0.7).

Subgroup analyses by treatment modality among continuous I-PSS and EPIC measures showed significant urinary function changes 6 months post-randomization in the surgery but not in the radiation group (Table 2). PC-PEP surgery patients had minimal changes in urinary symptoms and bother (IPSS change: 0.69, 95% CI: −2.1 to 3.5, *p* = 0.6; IPSS bother change: −0.24, 95% CI: −0.91 to 0.43, *p* = 0.5); in contrast, waitlist-control surgery patients experienced greater severity of urinary symptoms (change: −2.7, 95% CI: −5.30 to −0.13, *p* = 0.04) and bother (change: −1.3, 95% CI: −1.9 to −0.65, *p* < 0.001). The control group also reported more severe urinary incontinence and irritative/obstructive symptoms (EPIC change: 51, 95% CI: 42 to 60, *p* < 0.001; 6.9, 95% CI: 3.4 to 12, *p* < 0.001) compared to PC-PEP surgery patients (EPIC change: 24, 95% CI: 15 to 34, *p* < 0.001; −2.3, 95% CI: −7.7 to 3.1, *p* < 0.001). While the initial analysis did not yield a significant interaction between group and time, further post hoc analyses provided valuable insights (Figure 2). Both the surgery waitlist-control group (EPIC change: 47, 95% CI: 37 to 57, *p* < 0.001) and the PC-PEP group (EPIC change: 42, 95% CI: 32 to 53, *p* < 0.001) experienced a decline in reported sexual function from baseline to 6 months., as well as baseline to 12 months (change: 43, 95% CI: 34 to 53, *p* < 0.001; and change: 42, 95% CI: 32 to 53, *p* < 0.001). The analysis at the 6-month mark revealed that the surgery patients in the waitlist-control group reported a significantly greater decline in function compared to their counterparts in the surgery PC-PEP group (change: −12, 95% Confidence Interval [CI]: −23 to −1.7, *p* = 0.024). However, at the 12-month follow-up, this difference between the groups was not statistically significant (change: −8.9, 95% CI: −21 to 2.7, *p* = 0.13).

It is important to note that no significant interactions were observed in the treatment subgroup analyses among patients who underwent radiation therapy, as detailed in Table 2. The Supplementary Materials provides additional insights with pre- to post-intervention comparisons for both the early (those who started PC-PEP immediately post-randomization) and the late (those who commenced PC-PEP post the 6-month crossover) PC-PEP groups. We advise exercising caution in interpreting these results, especially considering that the late/waitlist-control intervention group initiated the PFMT after their primary treatment, whereas the early group began the PFMT prior to and during their treatment.

Two-level linear modeling analyses found a significant interaction between PC-PEP and waitlist-control groups from baseline to 12 months based on EPIC urinary incontinence scores (11, 95% CI: 4.1 to 19, *p* = 0.002). This effect was observed among surgery but not radiation patients (Supplementary Materials). At 12 months, despite no statistically significant differences at baseline, patients in the waitlist-control group exhibited worse urinary incontinence than the early PC-PEP group (−11, 95% CI: −17 to −4.5, *p* < 0.001), especially among surgery patients (−24, 95% CI: −33 to −14, *p* < 0.001). No other significant interactions were detected.

**Figure 3:**
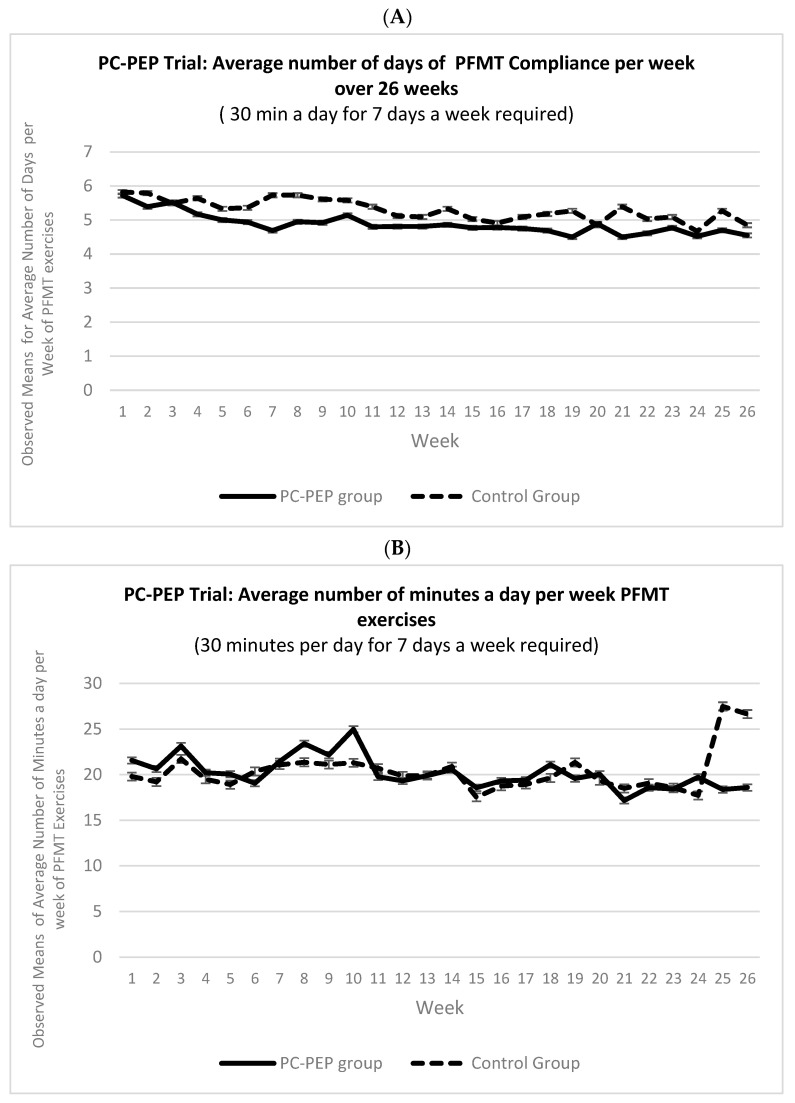
Observed means for (**A**) average weekly PFMT compliance and (**B**) average number of minutes per day of PEMF compliance between the control and PC-PEP groups over 26 weeks among 128 curative prostate cancer patients treated in Nova Scotia, Canada.

Figure 3 displays PFMT compliance over 26 weeks for early and late (waitlist-control) intervention groups, with an average of 4.9 to 5.3 days per week (70% to 76%) and 20 min of daily PFMT (76% compliance) (Supplementary Materials). 

Generalized linear mixed modeling assessed the early vs. late (waitlist-control) groups over 26 weeks, revealing no significant interactions (Table 3). 

A main effect of time was noted for the average weekly PFMT exercises, but follow-up analysis showed no significant trend (β = −0.27, t = −2.0, *p* = 0.052, OR = 0.97, 95% CI: 0.95–1.00).

## 4. Discussion

In this secondary analysis of the PC-PEP trial, early enrollment significantly improved self-reported urinary symptoms and continence in men scheduled for curative-intent prostate cancer surgery or radiotherapy, with notable benefits observed at both the 6-month and 12-month follow-up points. Subgroup analyses highlighted that the improvements were more prominent among surgery patients when compared to those receiving radiation treatment. The intervention did not significantly impact bowel or hormone-related self-reported function but did lead to improved sexual function in the surgery group, particularly in the early PC-PEP group at 6, but not at 12 months compared to the waitlist-control group. Importantly, compliance rates were not statistically significantly different between the early and late (waitlist-control) PC-PEP intervention groups.

Previous studies assessing PFMT’s effectiveness in improving urinary symptoms during prostate cancer treatment have yielded conflicting results, often due to short durations and poor compliance with prescribed PFMT [15]. Poor compliance with the prescribed program is a major factor contributing to the lack of PFMT effectiveness in some trials [15,16,17]. To maximize compliance in our PFMT program (7–9-min sessions required three times a day) for six months, participants received daily email reminders (with links to the daily PFMT instructional videos) and had the option to receive three daily text reminders (about two-thirds of the participants opted in). This comprehensive approach likely contributed to high compliance rates, with 70% of men reporting compliance, averaging about 20 min of PFMT per day, meeting 76% of the prescribed regimen. Notably, our extended six-month program duration underpins the evident benefits in self-reported urinary symptoms, surpassing the longest length (19 weeks) of reported PFMT trials to date [15,16,17,18].

Our study demonstrates that early enrollment in the PC-PEP program results in substantial improvements in sexual function and urinary continence among men undergoing prostate cancer surgery within the initial six months following diagnosis. Notably, although the differences between the intervention and control groups persist at the 12-month assessment, statistical significance diminishes for the sexual function outcome. Clinically, these findings emphasize the potential of PC-PEP to enhance the quality of life, especially for those undergoing surgery during the early post-diagnosis phase. This suggests a compelling rationale for considering the integration of PC-PEP into standard-of-care protocols.

At our institution, referring patients to a pelvic floor nurse for postoperative care is a standard procedure. Nonetheless, our analysis indicates a modest engagement rate, with only 32% of patients attending the recommended sessions—comprising 5 participants in the PC-PEP group and 15 in the waitlist-control group. This attendance rate falls significantly below our expectations, as referenced in the literature [31]. The reasons for this discrepancy are not fully understood due to a lack of direct evidence. Potential factors influencing patient participation may include logistical challenges such as scheduling conflicts and transportation difficulties, insufficient awareness or understanding of pelvic floor training benefits, or psychological barriers, including reluctance to engage in discussions about pelvic health. Additionally, stigma or discomfort associated with such treatments may deter patient involvement.

To enhance the effectiveness of our patient empowerment program, it is critical to not only provide specialized postoperative care but also ensure its accessibility and acceptability. Addressing barriers to care, improving patient education on the benefits of pelvic floor muscle training (PFMT), and adopting more adaptable or personalized approaches to care delivery are strategies worth considering. The significant improvements in urinary function and overall quality of life attributed to the PC-PEP intervention highlight its value and potential for incorporation into the standard postoperative regimen.

The low participation rate in PFMT sessions, despite their known advantages, points to the need for further investigation into patient engagement strategies. A deeper exploration into the reasons behind non-attendance and the experiences of patients could shed light on how to optimize postoperative care pathways and empowerment programs, thereby maximizing patient involvement and enhancing recovery outcomes. Our team is currently leading a Pan-Canadian and international implementation study to evaluate the wider clinical applicability of PC-PEP. This trial aims to explore personalized interventions for prostate cancer patients across various treatment spectrums, including active surveillance and metastatic disease, thereby contributing to a more nuanced understanding of patient needs and preferences.

This study is not without limitations. Importantly, this is a secondary analysis of data from the PC-PEP trial [4] and may simply represent a sampling error. Secondly, the primary outcome for this analysis, which is self-reported urinary symptoms, may also be biased by the improved mental health found in the intervention group. This could presumably lead to an under-reporting of actual symptoms or an increased capacity to tolerate side effects [4]. Thirdly, objective endpoints of urinary function, like the weight of incontinence pads, were not assessed in our trial. Nonetheless, the validated questionnaires used in this study (IPSS and EPIC) are widely used, both in research and in clinical practice. Lastly, long-term urinary outcomes past the year point for this trial are not available. However, a Phase 4 Pan-Canadian and International Implementation trial following participants over 2 years is underway.

The strength of the PFMT program in the 6-month PC-PEP program is notable. PC-PEP is relatively inexpensive to administer (costs approximately $200 CAN per patient) and easily administered intervention, accessible even to patients in remote areas, thereby including a sub-population of patients typically excluded from tertiary care PFMT programs. Importantly, the period between diagnosis and treatment provides a prime opportunity to activate the patient’s role in their own health care [32,33]. The anxiety men experience during this uncertain time can motivate them to adopt permanent lifestyle changes that may impact their long-term mental and physical health, in addition to potentially improving or speeding up the recovery of their urinary and sexual function after treatment [4,32,33].

The backbone of PC-PEP (six months of daily reminder emails and video teaching and encouragement) can also act as a platform to test other PFMT interventions or modifications of the PCPEP program [34]. Biofeedback, individualized training with PFMT experts, and penile rehabilitation for men undergoing prostatectomy may improve urinary symptoms even more effectively when paired with this multi-faceted program base and daily reminders. Testing the addition of video modules and interactive components may finally definitively prove the value of PFMT.

## 5. Conclusions

Our analysis underscores the substantial benefits of integrating Pelvic Floor Muscle Training (PFMT) into a six-month empowerment program for men undergoing prostate cancer treatment, especially those undergoing surgery. Prostate cancer treatment significantly impacts patients’ quality of life, with incontinence and sexual dysfunction being particularly distressing. Addressing these issues is essential for optimal patient care [7]. PFMT within our program, known as PC-PEP (Prostate Cancer-Patient Empowerment Program), not only benefits patients but also advances PFMT research. PC-PEP serves as a valuable platform for gathering data on PFMT’s effectiveness in managing urinary symptoms and improving the well-being of prostate cancer patients. This research contributes to the scientific knowledge base surrounding PFMT’s applications in prostate cancer care. PC-PEP’s simplicity, affordability, and adaptability make it a valuable resource for empowering men undergoing prostate cancer treatment, regardless of their location or access to healthcare resources.

Our study’s pivotal discovery is the demonstrable improvement in self-reported urinary symptoms and continence among participants of the Prostate Cancer-Patient Empowerment Program (PC-PEP), notably within the initial six months following treatment. This improvement is especially pronounced in patients undergoing surgery, highlighting the program’s particular efficacy in this subgroup. Furthermore, while PC-PEP’s influence on sexual function showed promising trends at six months, the differentiation from control diminished by the twelve-month mark. These findings underscore the potential of early, targeted interventions like PC-PEP in mitigating some of the most challenging side effects of prostate cancer treatment, namely urinary incontinence and sexual dysfunction. By providing a structured support system through PC-PEP, our research not only contributes to the clinical management of prostate cancer but also to the broader discourse on patient-centered care approaches that prioritize quality of life post-treatment.

## Figures and Tables

**Table 1:** Sample baseline characteristics comparison between the Prostate Cancer—Patient Empowerment Program (PC-PEP) intervention and control wait-list groups among 128 prostate cancer patients undergoing curative-intent treatment in Nova Scotia, Canada.

	PC-PEP Median (Quartile) n, % (n = 66)	Control Median (Quartile) n, % (n = 62)	*p*
*Urologic self-reported outcomes*			
IPSS ^a^ sum scores	66, 7 (3, 12)	62, 8 (4, 12)	0.3
IPSS ^a^ bother score	66, 2 (1, 4)	62, 2 (1, 3)	1.0
EPIC ^b^ Urinary Incontinence	66, 100 (62, 139)	62, 100 (74, 126)	0.7
EPIC ^b^ Urinary Irritative/Obstructive	66, 94 (85, 104)	62, 94 (85, 104)	0.4
EPIC ^b^ Bowel function	66, 100 (96,104)	62, 100 (96,104)	0.8
EPIC ^b^ Sexual function	66, 62 (37, 87)	62, 61 (37, 84)	0.9
EPIC ^b^ Hormonal function	66, 95 (89, 101)	62, 95 (88, 103)	0.5
*Demographic characteristics*			
Age (yr)	66, 66 (60, 70)	62, 68 (61, 72)	0.2
Body Mass Index	66, 29 (24, 34)	62, 27 (23, 31)	0.5
Household Income at baseline, >30,000 CAD/past year	54, 82%	52, 84%	0.5
Race, White	60, 91%	61, 98%	0.068
Education, university or above	31, 47%	37, 60%	0.16
Employed (part of full time)	22, 33%	23, 37%	0.7
Charlson Comorbidity Index	66, 2 (2, 3)	62, 3 (2, 3)	0.5
Self-identified as a cigarette smoker	5, 8%	3, 5%	0.7
*Diagnosis and treatment-relevant characteristics*			
Stage of cancer			
Risk Category (RP ^c^ + primary RT ^d^ ± TH ^e^) ^f^			0.6
Low	1, 1.5%	2, 3.2%	
Intermediate	42, 75%	40, 67%	
High	13, 23%	18, 30%	
PSA (ng/mL) at time of RT (salvage group only)	10, 0.11 (0.065, 0.16)	2, 0.28 (0.18, 0.37)	0.5
Prescribed ADT ^g^	27, 41%	21, 34%	0.4
Treatment modality			0.067
RP ^c^	29, 44%	33, 53%	
RT ^d^	27, 41%	27, 44%	
Salvage RT ^h^	10, 15%	2, 3.2%	
Nr. days between RP ^c^ and standard of care PFMT ^i^ appointment (referral)	5, 27 (12, 65)	15, 34 (18, 87)	0.4
No. of visits to the PFMT ^i^ nurse specialist at the hospital	5, 1 (1, 7)	15, 2 (1, 5)	0.9
Time between the first and last PFMT visit to the hospital (days)	5, 0 (0, 726)	15, 49 (0, 202)	0.7
Time between randomization and RP ^c^ or RT ^d^ treatment (days)	66, 61 (34, 99)	62, 73 (29, 101)	0.3
Intake of prescribed medication for depression, anxiety, or both at the time of entering the trial	12, 18%	7, 11%	0.3
Absence of cancer recurrence at 6 months post-randomization	63, 96%	58, 94%	0.6
*The specific reasons for patients’ visits to the Hospital’s PFMT ^i^ Nurse as part of their standard of care during the duration of the trial*			
PFMT ^i^ biofeedback	1	11	
PFMT ^i^ verbal education provided, trial of void, and PFMT ^i^ handout provided—single visit	3	3	
Removal of catheter and PFMT ^i^ provided—single visit	1	1	

Summary statistics are presented as n, median (quartiles), and n followed by percentage for categorical data. ^a^ I-PSS = International Prostate Symptom Score; ^b^ EPIC = Expanded Prostate Cancer Index Composite; ^c^ Radical prostatectomy; ^d^ Radiation therapy; ^e^ Hormone therapy; ^f^ National Comprehensive Cancer Network (NCCN); ^g^ ADT—Androgen deprivation therapy; ^h^ The Radiation therapy and salvage radiation groups were pooled together to allow for meaningful comparisons; ^i^ Pelvic Floor Muscle Training.

**Table 2:** Results of the two-level linear model analyses for the entire sample and subgroup analyses by treatment type (62 surgery; 66 radiation) fitting International Prostate Symptom Score (I-PSS) score, I-PSS Quality of Life score, Expanded Prostate Cancer Index Composite (EPIC) Urinary Incontinence, EPIC Urinary Irritative/Obstructive, EPIC-Bowel, Sexual and Hormonal self-reported function among 128 prostate cancer patients from Halifax, Nova Scotia, evaluating differences between groups (PC-PEP vs. waitlist control) from baseline to 6 months.

	I-PSS Sum Score			
*Level*	*Parameter Estimate*	*95% Confidence Interval*		*p*
		*Lower*	*Upper*	
Group (Control vs. PC-PEP)	2.8	0.58	4.9	0.013
Time (baseline vs. 6 months)	−0.076	−1.8	1.7	0.9
Time × Group (Control)	−2.4	−4.9	0.097	0.059
**Surgery**				
Group (Control vs. PC-PEP)	5.4	2.1	8.7	0.02
Time (baseline vs. 6 months)	0.69	−2.1	3.5	0.6
Time × Group (Control)	−3.4	−7.2	3.5	0.077
**Radiation**				
Group (Control vs. PC-PEP)	−0.28	−3.1	2.6	0.9
Time (baseline vs. 6 months)	−0.68	−3.0	1.6	0.6
Time × Group (Control)	−1.5	−5.0	1.9	0.4
	**IPSS Bother score**			
Group (Control vs. PC-PEP)	0.88	0.35	1.4	0.001
Time (baseline vs. 6 months)	−0.11	−0.51	0.30	0.6
Time × Group (PCPEP)	−0.88	−1.5	−0.29	0.004
**Surgery**				
Group (Control vs. PC-PEP)	1.8	1.02	2.5	<0.001
Time (baseline vs. 6 months)	−0.24	−0.91	0.43	0.5
Time × Group (Control)	−1.03	−1.9	0.12	0.028
**Radiation**				
Group (Control vs. PC-PEP)	−0.062	−0.76	0.63	0.9
Time (baseline vs. 6 months)	0.00	−0.50	0.50	1.0
Time × Group (Control)	−0.66	−1.4	0.10	0.089
	**EPIC Urinary Incontinence score**			
Group (Control vs. PC-PEP)	−18	−25	−11	<0.001
Time (baseline vs. 6 months)	11	3.9	17	0.002
Time × Group (Control)	20	10	29	<0.001
**Surgery**				
Group (Control vs. PC-PEP)	−31	−41	−22	<0.001
Time (baseline vs. 6 months)	25	15	34	<0.001
Time × Group (Control)	27	14	40	<0.001
**Radiation**				
Group (Control vs. PC-PEP)	−1.9	−9.8	6.05	0.6
Time (baseline vs. 6 months)	−0.34	−4.9	4.2	0.9
Time × Group (Control)	6.2	−0.65	13	0.075
	**EPIC Urinary Irritative/Obstruction score**			
Group (Control vs. PC-PEP)	−7.6	−12	−3.4	<0.001
Time (baseline vs. 6 months)	−1.4	−4.7	1.9	0.4
Time × Group (Control)	6.5	1.7	11	0.008
**Surgery**				
Group (Control vs. PC-PEP)	−15	−21	−8.3	<0.001
Time (baseline vs. 6 months)	−2.3	−7.7	−8.3	0.4
Time × Group (Control)	9.2	1.7	17	0.017
**Radiation**				
Group (Control vs. PC-PEP)	−0.45	−6.1	5.3	0.9
Time (baseline vs. 6 months)	−0.73	−4.91	3.45	0.7
Time × Group (Control)	3.7	−2.6	10	0.2
	**EPIC Bowel scores**			
Group (Control vs. PC-PEP)	−2.8	−6.7	1.2	0.17
Time (baseline vs. 6 months)	2.5	−0.30	5.3	0.079
Time × Group (Control)	2.03	−2.02	6.09	0.3
**Surgery**				
Group (Control vs. PC-PEP)	−2.7	−7.9	2.5	0.3
Time (baseline vs. 6 months)	−0.53	−3.2	2.1	0.7
Time × Group (Control)	2.1	−1.5	5.7	0.3
**Radiation**				
Group (Control vs. PC-PEP)	−3.1	−9.2	2.9	0.3
Time (baseline vs. 6 months)	4.9	0.28	9.6	0.038
Time × Group (Control)	3.04	−4.0	10	0.4
	**EPIC Sexual score**			
Group (Control vs. PC-PEP)	−2.7	−12	6.2	0.6
Time (baseline vs. 6 months)	30	23	37	<0.001
Time × Group (Control)	4.8	−5.7	15	0.4
**Surgery**				
Group (Control vs. PC-PEP)	−12	−23	−1.7	0.024
Time (baseline vs. 6 months)	43	32	54	<0.001
Time × Group (Control)	4.3	−10	19	0.6
**Radiation**				
Group (Control vs. PC-PEP)	10	−3.4	24	0.14
Time (baseline vs. 6 months)	20	11	29	<0.001
Time × Group (Control)	0.89	−12	14	0.9
	**EPIC Hormonal score**			
Group (Control vs. PC-PEP)	−2.08	−6.6	2.4	0.4
Time (baseline vs. 6 months)	4.6	1.4	7.7	0.005
Time × Group (Control)	1.3	−3.2	5.7	0.6
**Surgery**				
Group (Control vs. PC-PEP)	−5.9	−12	0.32	0.063
Time (baseline vs. 6 months)	−0.34	−4.5	3.9	0.9
Time × Group (Control)	3.7	−2.08	9.4	0.2
**Radiation**				
Group (Control vs. PC-PEP)	1.3	−5.1	7.7	0.7
Time (baseline vs. 6 months)	8.4	4.01	13	<0.001
Time × Group (Control)	0.24	−6.3	6.8	0.9

Note: Models included group, time (month), group × time, age, treatment modality (surgery vs. radiation), Charlson Comorbidity Index, and days between randomization and treatment.

**Table 3:** Generalized linear mixed modeling (GLMM) analyses evaluating the interaction between group (early versus late/waitlist-control PC-PEP intervention) and time (26 weeks) for PFMT compliance outcomes among 128 prostate cancer patients from Halifax, Nova Scotia, Canada.

Tests of Fixed Effects			
Average number of days of PFMF compliance per week			
Time	1	4.2	0.04
Group	1	2.0	0.16
Group × Time	1	1.08	0.3
Average number of days of PFMF compliance per week ^1^			
Time	1	3.9	0.052
Group	1	1.2	0.3
Average daily duration in minutes of PFMT compliance per week			
Time	1	0.27	0.6
Group	1	3.2	0.08
Group × Time	1	2	0.16

^1^ Follow-up analyses for significant main effects excluding the interaction. PC-PEP = Prostate Cancer—Patient Empowerment Program; PFMT = Pelvic Floor Muscle Training.

## Data Availability

Data from this study are available to researchers through a data access process in compliance with the Patient Privacy and Protection Research Act (NSHA Research Ethics Board).

## Supplementary Materials

The following supporting information can be downloaded at https://www.mdpi.com/article/10.3390/cancers16050958/s1, Table S1: PC-PEP PEMT weekly instructions and descriptors and Weekly Compliance Survey Questions for Assessing the Pelvic Floor Muscle Training (PFMT) Component of the Prostate Cancer-Physical Exercise Program (PC-PEP); Table S2: Results of the two-level linear model analyses for the entire sample and subgroup analyses by treatment type (62 surgery; 66 radiation) fitting International Prostate Symptom Score (I-PSS) score, I-PSS Quality of Life score, Expanded Prostate Cancer Index Composite (EPIC) Urinary Incontinence, EPIC Urinary Irritative/Obstructive, EPIC-Bowel, Sexual and Hormonal self-reported function among 128 prostate cancer patients from Halifax, Nova Scotia, evaluating differences between groups (PC-PEP vs. waitlist control) from baseline to 6 months (without covariates adjustment); Table S3: Results of the two-level linear model analyses fitting International Prostate Symptom Score (I-PSS) score, I-PSS Quality of Life score, Expanded Prostate Cancer Index Composite (EPIC) Urinary Incontinence, EPIC Urinary Irritative/Obstructive, EPIC-Bowel, Sexual and Hormonal self-reported function among prostate cancer patients evaluating differences between groups (PC-PEP vs. late PC-PEP for the waitlist control group) from baseline to 12 months; Table S4: PFMT compliance outcomes over 26 weeks for the early and late (waitlist-control) groups among 128 men from Halifax, Nova Scotia; Table S5: GLMM analysis evaluating group (early versus late PC-PEP intervention) × time (26 weeks) interaction for PFMT compliance among 128 prostate cancer patients from Halifax, Nova Scotia, Canada; Table S6: Displaying subgroup (treatment type) results of early (PC-PEP received at randomization) vs. late (PC-PEP received at 6 months) × time (pre-post intervention) analysis of outcomes.
