# Supplementary material for: Six-Month Prostate Cancer Empowerment Program (PC-PEP) Improves Urinary Function: A Randomized Trial
Source: Cancers (Basel). 2024 Feb 27;16(5):958. doi: 10.3390/cancers16050958 (PMC10930549; doi:10.3390/cancers16050958)
Supplement: Supplementary file 1 [file cancers-16-00958-s001.zip › cancers-2879010-supplementary.pdf]

# SUPPLEMENTAL MATERIALS

**Table S1:** PC-PEP PEMT weekly instructions and descriptors and Weekly Compliance Survey Questions for Assessing the Pelvic Floor Muscle Training (PFMT) Component of the Prostate Cancer-Physical Exercise Program (PC-PEP)

| *Week* | *Position* | *Start of Long Holds* | *End of Long Holds* | *Quick Flicks* | *Blow Before You Go Exercise* |
|---|---|---|---|---|---|
| 1 | Lying and sitting | Lying on your back, contract your pelvic floor muscles gradually increasing level of effort up to 100% over the 10 second period. | Relax fully | Sitting upright, fully contract the pelvic floor muscles, and then release immediately once peak contraction is achieved. Perform at a pace that works for your level of ability. Try to make transitions between contraction and relaxation as quick as possible. Repeat 10 times. | Sitting upright, breathe in, then breathe out and hold that breath. Then fully contract your pelvic floor muscles and stand up. Once standing release the contraction and breathe normally. Perform 1 repetition. |
| 2 | Sitting and standing | Sitting upright with a towel rolled underneath you, contract your pelvic floor muscles for 10 seconds, gradually increasing level of effort up to 100%. | Relax fully | Sitting upright, fully contract the pelvic floor muscles, and then release immediately once peak contraction is achieved. Perform at a pace that works for your level of ability. Try to make transitions between contraction and relaxation as quick as possible. Repeat 10 times. | Same as previous week |
| 3 | Lying and sitting | Lying on your back, contract your pelvic floor muscles gradually increasing level of effort up to 100% over the 10 second period. | First still lying down, fully relax and breathe out for 10 seconds once full contraction is achieved. Perform the same technique sitting upright for the second set | Lying on your back, fully contract the pelvic floor muscles, and then release immediately once peak contraction is achieved. Perform at a pace that works for your level of ability. Try to make transitions between contraction and relaxation as quick as possible. Perform the same technique sitting upright. Repeat 10 times for each. | Same as previous week |

| | | | | | |
|---|---|---|---|---|---|
| 4 | Sitting and standing | Sitting upright, contract your pelvic floor muscles for 10 seconds, gradually increasing level of effort by 25% up to 100%. Perform the same technique while standing upright. | Sitting upright, fully relax and breathe out for 10 seconds. Perform the same technique standing upright for the second set | Sitting upright, fully contract the pelvic floor muscles, and then release immediately once peak contraction is achieved. Perform at a pace that works for your level of ability. Try to make transitions between contraction and relaxation as quick as possible. Perform the same technique but standing upright. Repeat 10 times for each. | Same as previous week |
| 5 | Sitting and standing | Same as previous week | Sitting upright, fully relax and breathe out for 10 seconds. Perform the same technique standing upright for the second set | Sitting upright, fully contract the pelvic floor muscles, and then release immediately once peak contraction is achieved. Perform at a pace that works for your level of ability. Try to make transitions between contraction and relaxation as quick as possible. Perform the same technique but standing upright. Repeat 10 times for each. | Same as previous week |
| 6 | Sitting and standing | Sitting upright, contract your pelvic floor muscles for 10 seconds, gradually increasing level of effort by 25% up to 100% and maintaining that 100% till the end of the 10 seconds. | Sitting upright, gradually relax and breathe out for 5 seconds, decreasing effort by 25%, then fully relax. Perform the same technique standing upright for the second set | Sitting upright, fully contract the pelvic floor muscles, and then release immediately once peak contraction is achieved. Perform at a pace that works for your level of ability. Try to make transitions between contraction and relaxation as quick as possible. Repeat 10 times. | Same as previous week |
| 7 | Sitting and standing | Same as previous week | Sitting upright, gradually relax and breathe out for 5 seconds, decreasing effort by 25%, then fully relax. Perform the same technique standing upright for the second set | Sitting upright, fully contract the pelvic floor muscles, and then release immediately once peak contraction is achieved. Perform at a pace that works for your level of ability. Try to make transitions between contraction and relaxation as quick as possible. Repeat 10 times. | Same as previous week |

| | | | | | |
|---|---|---|---|---|---|
| 8 | Sitting and standing | Same as previous week | Sitting upright, gradually relax and breathe out for 5 seconds, decreasing effort by 25%, then fully relax. Perform the same technique standing upright for the second set | Sitting upright, fully contract the pelvic floor muscles, and then release immediately once peak contraction is achieved. Perform at a pace that works for your level of ability. Try to make transitions between contraction and relaxation as quick as possible. Repeat 10 times. | Same as previous week |
| 9 | Sitting and standing | Same as previous week | Relax fully | Sitting upright, fully contract the pelvic floor muscles, and then release immediately once peak contraction is achieved. Perform at a pace that works for your level of ability. Try to make transitions between contraction and relaxation as quick as possible. Repeat 10 times. | Same as previous week |
| 10 | Sitting and standing | Same as previous week | Gradually relax and breathe out for 5 seconds, decreasing effort by 25%, then fully relax. | Sitting upright, fully contract the pelvic floor muscles, and then release immediately once peak contraction is achieved. Perform at a pace that works for your level of ability. Try to make transitions between contraction and relaxation as quick as possible. Repeat 10 times. | Same as previous week |
| 11 | Sitting and standing | Sitting upright, start by building tension from your anus to the base of your penis. Then, fully contract your pelvic floor muscles for 10 seconds. | Relax fully | Sitting upright, fully contract the pelvic floor muscles, and then release immediately once peak contraction is achieved. Perform at a pace that works for your level of ability. Try to make transitions between contraction and relaxation as quick as possible. Repeat 10 times. | Same as previous week |

| | | | | | |
|---|---|---|---|---|---|
| 12 | Sitting and standing | Sitting upright, start by building tension, increasing effort by 25% from your anus to the base of your penis. Then, fully contract your pelvic floor muscles for 10 seconds. | Gradually release the tension in your pelvic floor starting from the base of your penis to your anus. Repeat 20 times. | Sitting upright, fully contract the pelvic floor muscles, and then release immediately once peak contraction is achieved. Perform at a pace that works for your level of ability. Try to make transitions between contraction and relaxation as quick as possible. Repeat 10 times. | Standing: Breathe out, hold breath, fully contract your pelvic floor, Lunge and reach down as if picking something up. Return to standing. Relax pelvic floor and breathe normally. Repeat on other leg. |
| 13 | Sitting and standing | Sitting upright, start by building tension, increasing effort by 25% from the base of your penis to your anus. Then, fully contract your pelvic floor muscles for 10 seconds. | Relax fully | Sitting upright, fully contract the pelvic floor muscles, and then release immediately once peak contraction is achieved. Perform at a pace that works for your level of ability. Try to make transitions between contraction and relaxation as quick as possible. Repeat 10 times. | Same as previous week |
| 14 | Sitting and standing | Sitting upright, start by building tension, increasing effort by 25% from the base of your penis to your anus. Then, fully contract your pelvic floor muscles for 10 seconds. | Relax fully | Sitting upright, fully contract the pelvic floor muscles, and then release immediately once peak contraction is achieved. Perform at a pace that works for your level of ability. Try to make transitions between contraction and relaxation as quick as possible. Repeat 10 times. | Same as previous week |
| 15 | Sitting and standing | Sitting upright, start by building tension, increasing effort by 25% from the base of your penis to your anus. Then, fully contract your pelvic floor muscles for 10 seconds. | Gradually release the tension in your pelvic floor starting from the anus to base of your penis. | Sitting upright, fully contract the pelvic floor muscles, and then release immediately once peak contraction is achieved. Perform at a pace that works for your level of ability. Try to make transitions between contraction and relaxation as quick as possible. Repeat 10 times. | Same as previous week |
| 16 | Standing | Standing up, quickly build tension up to 100% in the pelvic floor muscles and | Relax fully | Sitting upright, fully contract the pelvic floor muscles, and then release immediately once peak contraction is achieved. Perform | Same as previous week |

| | | | | | |
|---|---|---|---|---|---|
| | | hold that full contraction for 10 seconds. | | at a pace that works for your level of ability. Try to make transitions between contraction and relaxation as quick as possible. Repeat 10 times. | |
| 17 | Standing | Sitting upright, contract your pelvic floor muscles for 10 seconds, gradually increasing level of effort by 25% up to 100% and maintaining that 100% effort till the end of the 10 seconds. | Standing up, gradually relax and breathe out for 5 seconds, decreasing effort by 25%, then fully relax. | Sitting upright, fully contract the pelvic floor muscles, and then release immediately once peak contraction is achieved. Perform at a pace that works for your level of ability. Try to make transitions between contraction and relaxation as quick as possible. Repeat 10 times. | Standing: Breathe out, hold breath, fully contract your pelvic floor. Squat (with both legs). Return to standing. Relax pelvic floor and breathe normally. Repeat once. |
| 18 | Standing | Same as previous week | Standing up, gradually relax and breathe out for 5 seconds, decreasing effort by 25%, then fully relax. | Sitting upright, fully contract the pelvic floor muscles, and then release immediately once peak contraction is achieved. Perform at a pace that works for your level of ability. Try to make transitions between contraction and relaxation as quick as possible. Repeat 10 times. | Same as previous week |
| 19 | Standing | Standing up, start by building tension, increasing effort by 25% from your anus to the base of your penis. Then, fully contract your pelvic floor muscles for 10 seconds. | Relax fully | Sitting upright, fully contract the pelvic floor muscles, and then release immediately once peak contraction is achieved. Perform at a pace that works for your level of ability. Try to make transitions between contraction and relaxation as quick as possible. Repeat 10 times. | Standing: Breathe out, hold breath, fully contract your pelvic floor. Reach down to the right side as if picking something up of floor. Return to standing. Relax pelvic floor and breathe normally. Repeat on other side. |
| 20 | Standing | Standing up, start by building tension, increasing effort by 25% from your anus to the base of your penis. Then, fully contract your pelvic floor muscles for 10 seconds. | Gradually release the tension in your pelvic floor starting from the base of your penis to your anus. | Sitting upright, fully contract the pelvic floor muscles, and then release immediately once peak contraction is achieved. Perform at a pace that works for your level of ability. Try to make transitions between contraction | Standing: Breathe out, hold breathe, fully contract your pelvic floor. Bring your hands to your sides and pretend to cough, maintaining the contraction in the pelvic floor. Relax pelvic floor and breathe normally. Repeat once. |

| | | | | | |
|---|---|---|---|---|---|
| | | | | and relaxation as quick as possible. Repeat 10 times. | |
| 21 | Standing | Sitting upright, start by building tension, increasing effort by 25% from the base of your penis to your anus. Then, fully contract your pelvic floor muscles for 10 seconds. | Relax fully | Sitting upright, fully contract the pelvic floor muscles, and then release immediately once peak contraction is achieved. Perform at a pace that works for your level of ability. Try to make transitions between contraction and relaxation as quick as possible. Repeat 10 times. | Standing: Breathe out, hold breath, fully contract your pelvic floor. Squat (with both legs). Return to standing. Relax pelvic floor and breathe normally. Repeat once. |
| 22 | Standing | Same as previous week | Gradually release tension in the pelvic floor. | Sitting upright, fully contract the pelvic floor muscles, and then release immediately once peak contraction is achieved. Perform at a pace that works for your level of ability. Try to make transitions between contraction and relaxation as quick as possible. Repeat 10 times. | Standing: Breathe out, hold breathe, fully contract your pelvic floor. Bring your hands to your sides and pretend to cough, maintaining the contraction in the pelvic floor. Relax pelvic floor and breathe normally. Repeat once. |
| 23 | Standing | Standing up, perform two quick contractions and hold a 100% contraction in the pelvic floor muscles for 10 seconds. | Relax fully | Sitting upright, fully contract the pelvic floor muscles, and then release immediately once peak contraction is achieved. Perform at a pace that works for your level of ability. Try to make transitions between contraction and relaxation as quick as possible. Repeat 10 times. | Standing: Breathe out, hold breath, fully contract your pelvic floor. Squat (with both legs). Return to standing. Relax pelvic floor and breathe normally. Repeat once. |
| 24 | Standing | Standing up, perform two quick contractions and hold a 100% contraction in the pelvic floor muscles for 10 seconds. | Two quick contractions then release | Sitting upright, fully contract the pelvic floor muscles, and then release immediately once peak contraction is achieved. Perform at a pace that works for your level of ability. Try to make transitions between contraction | Standing: Breathe out, hold breathe, fully contract your pelvic floor. Bring your hands to your sides and pretend to cough, maintaining the contraction in the pelvic floor. Relax pelvic floor and breathe normally. Repeat once. |

| | | | | | |
|---|---|---|---|---|---|
| | | | | and relaxation as quick as possible. Repeat 10 times. | |
| 25 | Standing | Standing up, perform three contractions starting from the anus to base of the penis, relaxing in between the first two contractions. | One quick contraction then relax | Sitting upright, fully contract the pelvic floor muscles, and then release immediately once peak contraction is achieved. Perform at a pace that works for your level of ability. Try to make transitions between contraction and relaxation as quick as possible. Repeat 10 times. | Standing: Breathe out, hold breath, fully contract your pelvic floor, Lunge and reach down as if picking something up. Return to standing. Relax pelvic floor and breathe normally. Repeat on other leg. |
| 26 | Standing | Standing up, perform two quick contractions and hold a 100% contraction in the pelvic floor muscles for 10 seconds. | Relax fully | Sitting upright, fully contract the pelvic floor muscles, and then release immediately once peak contraction is achieved. Perform at a pace that works for your level of ability. Try to make transitions between contraction and relaxation as quick as possible. Repeat 10 times. | Standing: Breathe out, hold breathe, fully contract your pelvic floor. Bring your hands to your sides and pretend to cough, maintaining the contraction in the pelvic floor. Relax pelvic floor and breathe normally. Repeat once. |

Note: Patients were asked to perform Kegel exercises three times daily, with each session lasting between 7 and 9 minutes. The average duration of a session was 8 minutes and 46 seconds, resulting in a total daily commitment of approximately 26.31 minutes.

---

Weekly Compliance Survey Questions for Assessing the Pelvic Floor Muscle Training (PFMT) Component of the Prostate Cancer-Physical Exercise Program (PC-PEP): During the PC-PEP Intervention/Training with our Pelvic Floor Nurse and in our daily message and weekly Kegels videos, we encouraged you to do pelvic floor (KEGELS) exercise routines, 3 times a day; for 7-9 minutes each time.

HOW MANY DAYS THIS WEEK, did you do the PELVIC FLOOR (KEGEL) EXERCISES that you were prescribed by the Study's Physiotherapist/or Pelvic Floor Nurse?

- ◯ not at all
- ◯ 1 day
- ◯ 2 days
- ◯ 3 days
- ◯ 4 days
- ◯ 5 days
- ◯ 6 days
- ◯ 7 days

---

On average, HOW LONG (in minutes) did you spend PER DAY, during THIS WEEK, doing KEGELS?

**Table** S2 Results of the two-level linear model analyses for the entire sample and subgroup analyses by treatment type (62 surgery; 66 radiation) fitting International Prostate Symptom Score (I-PSS) score, I-PSS Quality of Life score, Expanded Prostate Cancer Index Composite (EPIC) Urinary Incontinence, EPIC Urinary Irritative/Obstructive, EPIC-Bowel, Sexual and Hormonal self-reported function among 128 prostate cancer patients from Halifax, Nova Scotia, evaluating differences between groups (PC-PEP vs. waitlist control) from baseline to 6 months (without covariates adjustment)

| | **I-PSS Sum score** | | | |
|---|---|---|---|---|
| *Level* | *Parameter Estimate* | *95% Confidence Interval* | | *p* |
| | | *Lower* | *Upper* | |
| Group (Control vs. PC-PEP) | 3.1 | 0.93 | 5.3 | 0.005 |
| Time (baseline vs. 6 months) | -0.076 | -1.8 | 1.7 | 0.9 |
| Time x Group (Control) | -2.4 | -4.9 | 0.097 | 0.059 |
| | **IPSS Bother score** | | | |
| Group (Control vs. PC-PEP) | 0.96 | 0.43 | 1.5 | <0.001 |
| Time (baseline vs. 6 months) | -0.11 | -0.51 | 0.30 | 0.6 |
| Time x Group (PCPEP) | -0.88 | -1.5 | -0.29 | 0.004 |
| | **EPIC Urinary Incontinence score** | | | |
| Group (Control vs. PC-PEP ) | -20 | -28 | -12 | <0.001 |
| Time (baseline vs. 6 months) | 11 | 3.9 | 17 | 0.002 |
| Time x Group (Control) | 20 | 10 | 29 | <0.001 |
| | **EPIC Urinary Irritative/Obstruction score** | | | |
| Group (Control vs. PC-PEP ) | -7.8 | -12 | -3.5 | <0.001 |
| Time (baseline vs. 6 months) | -1.4 | -4.7 | 1.9 | 0.406 |
| Time x Group (Control) | 6.5 | 1.7 | 11 | 0.008 |
| | **EPIC Bowel scores** | | | |
| Group (Control vs. PC-PEP ) | -2.5 | -6.4 | 1.4 | 0.2 |
| Time (baseline vs. 6 months) | 2.5 | -0.30 | 5.3 | 0.079 |
| Time x Group (Control) | 2.03 | -2.02 | 6.09 | 0.3 |
| | **EPIC Sexual score** | | | |
| Group (Control vs. PC-PEP ) | -4.3 | -13 | 4.6 | 0.4 |
| Time (baseline vs. 6 months) | 30 | 23 | 37 | <0.001 |
| Time x Group (Control) | 4.8 | -5.7 | 15 | 0.4 |
| | **EPIC Hormonal score** | | | |
| Group (Control vs. PC-PEP ) | -1.7 | -6.1 | 2.7 | 0.5 |
| Time (baseline vs. 6 months) | 4.6 | 1.4 | 7.7 | 0.005 |
| Time x Group (Control) | 1.3 | -3.2 | 5.7 | 0.6 |

**Table S3** Results of the two-level linear model analyses fitting International Prostate Symptom Score (I-PSS) score, I-PSS Quality of Life score, Expanded Prostate Cancer Index Composite (EPIC) Urinary Incontinence, EPIC Urinary Irritative/Obstructive, EPIC-Bowel, Sexual and Hormonal self-reported function among prostate cancer patients evaluating differences between groups (PC-PEP vs. late PC-PEP for the waitlist control group) from baseline to 12 months.

| | **I-PSS Sum score** | | | |
|---|---|---|---|---|
| *Level* | *Parameter Estimate* | *95% Confidence Interval* | | *p* |
| | | *Lower* | *Upper* | |
| Group (Control vs. PC-PEP) | 2.01 | -0.36 | 4.09 | 0.057 |
| Time (baseline vs. 6 months) | 0.94 | -0.68 | 2.56 | 0.3 |
| Time x Group (Control) | -1.5 | -3.8 | 0.87 | 0.2 |
| **Surgery** | | | | |
| Group (Control vs. PC-PEP) | 3.9 | 0.69 | 7.01 | 0.017 |
| Time (baseline vs. 6 months) | 1.2 | -1.5 | 3.9 | 0.4 |
| Time x Group (Control) | -1.5 | -5.1 | 2.2 | 0.4 |
| **Radiation** | | | | |
| Group (Control vs. PC-PEP) | 0.013 | -2.8 | 2.8 | 0.9 |
| Time (baseline vs. 6 months) | 0.72 | -1.3 | 2.8 | 0.5 |
| Time x Group (Control) | -1.6 | -4.6 | 1.5 | 0.4 |
| | **IPSS Bother score** | | | |
| Group (Control vs. PC-PEP) | 0.49 | -0.015 | 1.01 | 0.057 |
| Time (baseline vs. 6 months) | -0.046 | -0.45 | 0.36 | 0.8 |
| Time x Group (PCPEP) | -0.47 | -1.05 | 0.11 | 0.11 |
| **Surgery** | | | | |
| Group (Control vs. PC-PEP) | 1.5 | 0.73 | 2.27 | <0.001 |
| Time (baseline vs. 6 months) | 0 | -0.69 | 0.69 | 1 |
| Time x Group (Control) | -0.73 | -1.7 | 0.22 | 0.13 |
| **Radiation** | | | | |
| Group (Control vs. PC-PEP) | -0.51 | -1.2 | 0.14 | 0.12 |
| Time (baseline vs. 6 months) | -0.081 | -0.56 | 0.402 | 0.74 |
| Time x Group (Control) | -0.19 | -0.92 | 0.53 | 0.6 |
| | **EPIC Urinary Incontinence score** | | | |
| Group (Control vs. PC-PEP ) | -11 | -17 | -4.5 | <0.001 |
| Time (baseline vs. 6 months) | 5.7 | 0.67 | 11 | 0.027 |
| Time x Group (Control) | 11 | 4.1 | 19 | 0.002 |
| **Surgery** | | | | |
| Group (Control vs. PC-PEP) | -22 | -32 | -12 | <0.001 |
| Time (baseline vs. 6 months) | 13 | 4.2 | 21 | 0.004 |
| Time x Group (Control) | 17 | 5.6 | 28 | 0.004 |
| **Radiation** | | | | |
| Group (Control vs. PC-PEP) | 2.2 | -5.3 | 9.7 | 0.6 |
| Time (baseline vs. 6 months) | 0.27 | -3.7 | 4.2 | 0.9 |
| Time x Group (Control) | 2.5 | -3.6 | 8.5 | 0.4 |
| | **EPIC Urinary Irritative/Obstruction score** | | | |

| | | | | |
|---|---|---|---|---|
| Group (Control vs. PC-PEP ) | -4.2 | -8.5 | 0.11 | 0.057 |
| Time (baseline vs. 6 months) | -2.2 | -5.7 | 1.4 | 0.2 |
| Time x Group (Control) | 2.6 | -2.5 | 7.7 | 0.3 |
| **Surgery** | | | | |
| Group (Control vs. PC-PEP) | -9.2 | -15 | -3.3 | 0.003 |
| Time (baseline vs. 6 months) | -3.1 | -8.5 | 2.3 | 0.3 |
| Time x Group (Control) | 3.4 | -3.9 | 11 | 0.4 |
| **Radiation** | | | | |
| Group (Control vs. PC-PEP) | 1.5 | -4.8 | 7.7 | 0.6 |
| Time (baseline vs. 6 months) | -1.4 | -6.3 | 3.5 | 0.6 |
| Time x Group (Control) | 2.01 | -5.3 | 9.3 | 0.6 |
| | **EPIC Bowel scores** | | | |
| Time x Group (Control) | -1.5 | -6.1 | 3.05 | 0.5 |
| Time (baseline vs. 6 months) | 5.3 | 1.7 | 8.9 | 0.004 |
| Time x Group (Control) | 0.67 | -4.5 | 5.9 | 0.8 |
| **Surgery** | | | | |
| Group (Control vs. PC-PEP) | -3.1 | -9.3 | 2.9 | 0.3 |
| Time (baseline vs. 6 months) | 1.05 | -2.7 | 4.8 | 0.6 |
| Time x Group (Control) | 2.5 | -2.7 | 7.7 | 0.3 |
| **Radiation** | | | | |
| Group (Control vs. PC-PEP) | -0.105 | -7.06 | 6.9 | 0.9 |
| Time (baseline vs. 6 months) | 8.6 | 2.8 | 14.5 | 0.004 |
| Time x Group (Control) | 0.042 | -8.8 | 8.8 | 0.9 |
| | **EPIC Sexual score** | | | |
| Group (Control vs. PC-PEP ) | -1.9 | -11 | 7.1 | 0.7 |
| Time (baseline vs. 6 months) | 30 | 23 | 37 | <0.001 |
| Time x Group (Control) | 3.7 | -6.5 | 14 | 0.5 |
| **Surgery** | | | | |
| Group (Control vs. PC-PEP) | -8.9 | -20 | 2.7 | 0.13 |
| Time (baseline vs. 6 months) | 42 | 32 | 53 | <0.001 |
| Time x Group (Control) | 0.99 | -13 | 15 | 0.9 |
| **Radiation** | | | | |
| Group (Control vs. PC-PEP) | 8.005 | -5.6 | 22 | 0.3 |
| Time (baseline vs. 6 months) | 20 | 11 | 29 | <0.001 |
| Time x Group (Control) | 2.3 | -11 | 16 | 0.7 |
| | **EPIC Hormonal score** | | | |
| Group (Control vs. PC-PEP ) | 0.56 | -3.8 | 4.9 | 0.8 |
| Time (baseline vs. 6 months) | 5.5 | 2.2 | 8.9 | 0.001 |
| Time x Group (Control) | -1.7 | -6.5 | 3.2 | 0.5 |
| **Surgery** | | | | |
| Group (Control vs. PC-PEP) | -3.04 | -8.8 | 2.7 | 0.3 |
| Time (baseline vs. 6 months) | 1.7 | -2.8 | 6.3 | 0.5 |

| | | | | |
|---|---|---|---|---|
| Time x Group (Control) | 0.25 | -5.9 | 6.5 | 0.9 |
| **Radiation** | | | | |
| Group (Control vs. PC-PEP) | 4.3 | -2.1 | 11 | 0.2 |
| Time (baseline vs. 6 months) | 8.5 | 3.6 | 13 | <0.001 |
| Time x Group (Control) | -2.5 | -9.8 | 4.9 | 0.5 |

Note: Models included group, time (month), group x time, age, treatment modality (surgery vs. radiation), Charlson Comorbidity Index, days between randomization and treatment.

**Table S4** PFMT compliance outcomes over 26 weeks for the early and late (waitlist-control) groups among 128 men from Halifax, Nova Scotia.

| **Early PC-PEP Group: Intervention received from the start of the trial (time of scheduled treatment) to 6 months** | | | | | | | | | | | | | | | | | | | | | | | | | | | |
|---|---|---|---|---|---|---|---|---|---|---|---|---|---|---|---|---|---|---|---|---|---|---|---|---|---|---|---|
| **Week number** | **1-26 M (SE)** | **1** M (SE) | **2** M (SE) | **3** M (SE) | **4** M (SE) | **5** M (SE) | **6** M (SE) | **7** M (SE) | **8** M (SE) | **9** M (SE) | **10** M (SE) | **11** M (SE) | **12** M (SE) | **13** M (SE) | **14** M (SE) | **15** M (SE) | **16** M (SE) | **17** M (SE) | **18** M (SE) | **19** M (SE) | **20** M (SE) | **21** M (SE) | **22** M (SE) | **23** M (SE) | **24** M (SE) | **25** M (SE) | **26** M (SE) |
| Nr. of days/ week | **4.87 (0.22)** | 5.72 (0.23) | 5.39 (0.28) | 5.52 (0.28) | 5.17 (0.30) | 5.00 (0.33) | 4.94 (0.30) | 4.69 (0.31) | 4.95 (0.30) | 4.92 (0.29) | 5.14 (0.27) | 4.80 (0.30) | 4.81 (0.31) | 4.81 (0.31) | 4.86 (0.32) | 4.77 (0.32) | 4.78 (0.28) | 4.75 (0.31) | 4.69 (0.32) | 4.50 (0.32) | 4.89 (0.30) | 4.50 (0.32) | 4.61 (0.32) | 4.77 (0.32) | 4.52 (0.32) | 4.70 (0.31) | 4.55 (0.32) |
| Avg. min/ day | **20.19 (1.00)** | 21.56 (1.56) | 20.63 (1.43) | 23.14 (1.46) | 20.21 (1.42) | 20.06 (1.69) | 19.06 (1.56) | 21.43 (2.24) | 23.39 (2.00) | 22.15 (1.62) | 24.98 (3.24) | 19.76 (1.50) | 19.31 (1.52) | 19.89 (1.49) | 20.55 (1.42) | 18.56 (1.49) | 19.31 (1.33) | 19.39 (1.51) | 21.09 (1.81) | 19.57 (1.59) | 20.05 (1.51) | 17.17 (1.52) | 18.58 (1.44) | 18.42 (1.60) | 19.72 (1.50) | 18.37 (1.35) | 18.59 (1.50) |
| **Late PC-PEP Group (waitlist control): intervention received from 6-12 months** | | | | | | | | | | | | | | | | | | | | | | | | | | | |
| Nr. of days/ week | **5.29 (0.35)** | 5.82 (0.36) | 5.79 (0.37) | 5.49 (0.38) | 5.64 ( 0.40) | 5.33 (0.43) | 5.36 (0.45) | 5.73 (0.35) | 5.73 (0.39) | 5.61 (0.38) | 5.58 (0.41) | 5.39 (0.42) | 5.12 (0.41) | 5.09 (0.44) | 5.33 (0.40) | 5.03 (0.45) | 4.91 (0.47) | 5.09 (0.43) | 5.18 (0.44) | 5.27 (0.41) | 4.85 (0.43) | 5.39 (0.37) | 5.03 (0.41) | 5.09 (0.43) | 4.67 (0.43) | 5.27 (0.41) | 4.85 (0.45) |
| Avg. min/ day | **20.35 (1.72)** | 19.79 (2.02) | 19.21 (2.07) | 21.73 (1.85) | 19.49 (1.90) | 18.91 (2.09) | 20.36 (2.16) | 21.06 (1.70) | 21.36 (1.99) | 21.11 (1.95) | 21.30 (2.13) | 20.71 (2.91) | 19.86 (1.92) | 19.91 (1.99) | 20.89 (1.85) | 17.52 (1.99) | 18.74 (2.03) | 18.92 (1.74) | 19.64 (2.16) | 21.34 (2.07) | 19.33 (1.93) | 18.49 (1.66) | 19.07 (1.83) | 18.58 (1.78) | 17.73 (1.82) | 27.48 (9.35) | 26.64 (9.42) |

[a]PFMT = Pelvic Floor Muscle Training

Table S5 GLMM analysis evaluating group (early versus late PC-PEP intervention) x time (26 weeks) interaction for PFMT compliance among 128 prostate cancer patients from Halifax, Nova Scotia, Canada.

| *TESTS OF FIXED EFFECTS* | | | |
|---|---|---|---|
| *Effect* | *df* | *F value* | *P* |
| PELVIC FLOOR/KEGELS EXERCISES - Average number of days completed per week | | | |
| Time | 1 | 4.21 | **0.04** |
| Group | 1 | 1.96 | 0.16 |
| Group x Time | 1 | 1.08 | 0.30 |
| PELVIC FLOOR/KEGELS EXERCISES - Average number of days completed per week[1] | | | |
| Time | 1 | 3.89 | 0.052 |
| Group | 1 | 1.19 | 0.28 |
| PELVIC FLOOR/KEGELS EXERCISES - Average number of minutes completed per week | | | |
| Time | 1 | 0.27 | 0.61 |
| Group | 1 | 3.19 | 0.08 |
| Group x Time | 1 | 2.00 | 0.16 |

[1]Follow up analyses for significant main effects in the absence of a significant interaction

**Table S6 Displaying subgroup (treatment modality) results of early (PC-PEP received at randomization) vs. late (PC-PEP received at 6 months) x time (pre-post intervention) analysis of outcomes.** This table presents the results of treatment subgroup analyses, comparing outcomes between patients receiving the Prostate Cancer-Physical Exercise Program (PC-PEP) at randomization (early intervention) versus those starting the program at 6 months (late intervention). Outcomes are measured pre- and post-intervention using a two-level linear model by treatment modality. The analysis includes the International Prostate Symptom Score (I-PSS), I-PSS Quality of Life score, and Expanded Prostate Cancer Index Composite (EPIC) scores for Urinary Incontinence, Urinary Irritative/Obstructive symptoms, Bowel, Sexual, and Hormonal functions. This evaluation assesses differences in self-reported function among prostate cancer patients, comparing those in the PC-PEP group with a waitlist control group.

| | **I-PSS Sum score** | | | |
|---|---|---|---|---|
| *Level* | *Parameter Estimate* | *95% Confidence Interval* | | *p* |
| | | *Lower* | *Upper* | |
| **Surgery** | | | | |
| Group (Late PC-PEP Waitlist-Control vs. early PC-PEP) | 2.9 | -0.36 | 6.3 | 0.081 |
| Time (pre vs. post-intervention) | 0.69 | -1.6 | 2.9 | 0.6 |
| Time x Group (Late PC-PEP Waitlist-Control) | 1.8 | -1.3 | 4.9 | 0.3 |
| **Radiation** | | | | |
| Group (Late PC-PEP Waitlist-Control vs. early PC-PEP) | -1.2 | -4.1 | 1.8 | 0.4 |
| Time (pre vs. post-intervention) | -0.68 | -2.8 | 1.5 | 0.5 |
| Time x Group (Late PC-PEP Waitlist-Control) | 2.05 | -1.2 | 5.3 | 0.2 |
| | **IPSS Bother score** | | | |
| **Surgery** | | | | |
| Group (Late PC-PEP Waitlist-Control vs. early PC-PEP) | 1.2 | 0.49 | 2.01 | 0.001 |
| Time (pre vs. post-intervention) | -0.24 | -0.79 | 0.31 | 0.4 |
| Time x Group (Late PC-PEP Waitlist-Control) | 0.79 | -0.79 | 0.31 | 0.4 |
| **Radiation** | | | | |
| Group (Late PC-PEP Waitlist-Control vs. early PC-PEP) | -0.42 | -1.1 | 0.27 | 0.2 |
| Time (pre vs. post-intervention) | 0.00 | -0.49 | 0.49 | 1.0 |
| Time x Group (Late PC-PEP Waitlist-Control) | 0.37 | -0.36 | 1.12 | 0.3 |
| | **EPIC Urinary Incontinence score** | | | |
| **Surgery** | | | | |
| Group (Late PC-PEP Waitlist-Control vs. early PC-PEP) | -9.3 | -21 | 2.1 | 0.11 |
| Time (pre vs. post-intervention) | 25 | 16 | 33 | <0.001 |
| Time x Group (Late PC-PEP Waitlist-Control) | -46 | -58 | -35 | <0.001 |
| **Radiation** | | | | |
| Group (Late PC-PEP Waitlist-Control vs. early PC-PEP) | 0.96 | -6.9 | 8.8 | 0.8 |
| Time (pre vs. post-intervention) | -0.34 | -4.7 | 4.03 | 0.9 |
| Time x Group (Late PC-PEP Waitlist-Control) | -2.8 | -9.4 | 3.8 | 0.4 |
| | **EPIC Urinary Irritative/Obstruction score** | | | |
| **Surgery** | | | | |
| Group (Late PC-PEP Waitlist-Control vs. early PC-PEP) | -7.9 | -15 | -1.2 | 0.021 |
| Time (pre vs. post-intervention) | -2.3 | -7.7 | 2.5 | 0.4 |
| Time x Group (Late PC-PEP Waitlist-Control) | -4.4 | -11 | 2.2 | 0.2 |

| | | | | |
|---|---|---|---|---|
| **Radiation** | | | | |
| Group (Late PC-PEP Waitlist-Control vs. early PC-PEP) | 1.6 | -4.1 | 7.3 | 0.6 |
| Time (pre vs. post-intervention) | -0.73 | -4.91 | 3.45 | 0.7 |
| Time x Group (Late PC-PEP Waitlist-Control) | -1.6 | -7.9 | 4.7 | 0.6 |
| | **EPIC Bowel scores** | | | |
| **Surgery** | | | | |
| Group (Late PC-PEP Waitlist-Control vs. early PC-PEP) | -4.9 | -11 | 0.71 | 0.089 |
| Time (pre vs. post-intervention) | -0.53 | -4.03 | 2.9 | 0.8 |
| Time x Group (Late PC-PEP Waitlist-Control) | 2.6 | -2.2 | 7.4 | 0.3 |
| **Radiation** | | | | |
| Group (Late PC-PEP Waitlist-Control vs. early PC-PEP) | -3.7 | -11 | 3.2 | 0.29 |
| Time (pre vs. post-intervention) | 4.9 | 1.1 | 8.7 | 0.012 |
| Time x Group (Late PC-PEP Waitlist-Control) | -4.2 | -9.9 | 1.6 | 0.15 |
| | **EPIC Sexual score** | | | |
| **Surgery** | | | | |
| Group (Late PC-PEP Waitlist-Control vs. early PC-PEP) | -9.6 | -19 | -0.48 | 0.039 |
| Time (pre vs. post-intervention) | 43 | 34 | 51 | <0.001 |
| Time x Group (Late PC-PEP Waitlist-Control) | -46 | -58 | -35 | <0.001 |
| **Radiation** | | | | |
| Group (Late PC-PEP Waitlist-Control vs. early PC-PEP) | 9.4 | -3.9 | 23 | 0.16 |
| Time (pre vs. post-intervention) | 20 | 12 | 28 | <0.001 |
| Time x Group (Late PC-PEP Waitlist-Control) | -19 | -30 | -7.5 | 0.002 |
| | **EPIC Hormonal score** | | | |
| **Surgery** | | | | |
| Group (Late PC-PEP Waitlist-Control vs. early PC-PEP) | -4.8 | -12 | 1.9 | 0.161 |
| Time (pre vs. post-intervention) | -0.34 | -4.4 | 3.8 | 0.9 |
| Time x Group (Late PC-PEP Waitlist-Control) | -1.02 | -6.6 | 4.6 | 0.7 |
| **Radiation** | | | | |
| Group (Late PC-PEP Waitlist-Control vs. early PC-PEP) | 3.7 | -3.4 | 11 | 0.31 |
| Time (pre vs. post-intervention) | 8.4 | 3.8 | 13 | <0.001 |
| Time x Group (Late PC-PEP Waitlist-Control) | -11 | -18 | -4.02 | 0.002 |

Note: Models included group, time (month), group x time, age, treatment modality (surgery vs. radiation) – in the shole sample analysis, Charlson Comorbidity Index, days between randomization and treatment.
